# CD9 Counteracts Liver Steatosis and Mediates GCGR Agonist Hepatic Effects

**DOI:** 10.1002/advs.202400819

**Published:** 2024-06-05

**Authors:** Yi Zheng, Yuren Wang, Xin Xiong, Linlin Zhang, Jiaran Zhu, Bangliang Huang, Xiufei Liu, Jinbo Liu, Zhiming Zhu, Gangyi Yang, Hua Qu, Hongting Zheng

**Affiliations:** ^1^ Department of Endocrinology Translational Research of Diabetes Key Laboratory of Chongqing Education Commission of China the Second Affiliated Hospital of Army Medical University Chongqing 400037 China; ^2^ Department of Endocrinology Qilu Hospital of Shandong University Jinan 250012 China; ^3^ Department of Hypertension and Endocrinology the Third Affiliated Hospital of Army Medical University Chongqing 400042 China; ^4^ Department of Endocrinology the Second Affiliated Hospital of Chongqing Medical University Chongqing 400010 China

**Keywords:** glucagon receptor signaling, hepatic steatosis, lipid metabolism, tetraspanin CD9

## Abstract

Glucagon receptor (GCGR) agonism offers potentially greater effects on the mitigation of hepatic steatosis. However, its underlying mechanism is not fully understood. Here, it screened tetraspanin CD9 might medicate hepatic effects of GCGR agonist. CD9 is decreased in the fatty livers of patients and upregulated upon GCGR activation. Deficiency of CD9 in the liver exacerbated diet‐induced hepatic steatosis via complement factor D (CFD) regulated fatty acid metabolism. Specifically, CD9 modulated hepatic fatty acid synthesis and oxidation genes through regulating CFD expression via the ubiquitination‐proteasomal degradation of FLI1. In addition, CD9 influenced body weight by modulating lipogenesis and thermogenesis of adipose tissue through CFD. Moreover, CD9 reinforcement in the liver alleviated hepatic steatosis, and blockage of CD9 abolished the remission of hepatic steatosis induced by cotadutide treatment. Thus, CD9 medicates the hepatic beneficial effects of GCGR signaling, and may server as a promising therapeutic target for hepatic steatosis.

## Graphical abstract

Current study revealed that hepatic deficiency of CD9 exacerbated diet‐induced liver steatosis via complement factor D regulated fatty acid metabolism. Hepatic CD9 reinforcement alleviated liver steatosis, and blockage of CD9 abolished the remission of hepatic steatosis induced by cotadutide treatment. CD9 medicates the hepatic beneficial effects of GCGR signaling, and may server as a promising therapeutic target for hepatic steatosis.

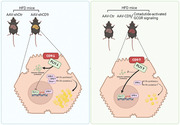

## 1 Introduction

Metabolic dysfunction‐associated steatotic liver disease (MASLD), characterized by excessive accumulation of lipid in hepatocytes, has emerged as the most common liver disease worldwide, and becomes the main cause of chronic liver disease and hepatocellular carcinoma.^[^ ^1^, ^2^ ^]^ The overall prevalence of MASLD is estimated to be 25–32.4%, usually paralleling the prevalence of obesity and type 2 diabetes.^[^ ^2^, ^3^, ^4^ ^]^ Although steady progress has been made in clarifying its pathogenesis and identifying therapeutic targets, no pharmacological therapies are approved.^[^ ^5^ ^]^ Recently, a couple of agents under clinical evaluation have gained great attention. Obeticholic acid and the fibroblast growth factor 21 analog pegozafermin led to improvements in liver fibrosis in randomized trials.^[^ ^6^, ^7^ ^]^ Single‐molecule agonists with dual or triple activity against the glucagon receptor (GCGR), glucagon‐like peptide‐1 receptor (GLP1R) and glucose‐dependent insulinotropic polypeptide receptor (GIPR) offered potentially greater effects of the reduction of hepatic steatosis and body weight,^[^ ^8^, ^9^, ^10^ ^]^ wherein their action to reduce hepatic lipid accumulation were proved mainly through GCGR signaling.^[^ ^11^, ^12^, ^13^ ^]^

GCGR is a transmembrane receptor which belongs to the secretin‐like class B family of G‐protein‐coupled receptors, and its agonist showed impressive effects on regulating hepatic lipid control and energy expenditure in a series of rodent models.^[^ ^12^, ^13^ ^]^ A GCGR and GLP1R dual agonist cotadutide (Cot), which was previously reported to reduce hepatic steatosis in people with type 2 diabetes mellitus (T2DM),^[^ ^14^ ^]^ has been demonstrated its action on the liver to reduce lipid content through GCGR agonism, and extrahepatic effects was via GlP1R engagement.^[^ ^11^ ^]^ A very recent two‐part, randomized phase 2a trial of Cot provides evidence of additional benefits could be attributed to GCGR engagement for improving metabolic health of the human liver.^[^ ^15^ ^]^ Thus, GCGR agonist is a promising therapeutic option for the treatment of liver steatosis, while the underlying mechanism of GCGR activation in treating MASLD remains unclear.

Tetraspanins are a family of cell surface glycoproteins featured by four transmembrane domains, each with characteristic structural features, including a conserved CCG motif in the large extracellular loop. Owing to the structure, tetraspanins involve in a number of normal and pathological processes, and an abundance of recent evidence suggests that targeting tetraspanins should be therapeutically beneficial.^[^ ^16^ ^]^ For example, CD151 supports a variety of primary tumor growth, metastasis and angiogenesis, while anti‐CD151 antibodies have been shown to inhibit spontaneous and experimental metastasis and tumor cell intravasation.^[^ ^17^ ^]^ In hepatocytes, CD81 is considered as an attractive target since it is vital to the binding and infection of hepatitis C virus.^[^ ^18^ ^]^ As for tetraspanin CD9, it is widely expressed in organs and cells, and it has been primarily proved as a tumor suppressor.^[^ ^19^ ^]^ In this study, we identify a novel role of CD9 to combat liver steatosis, and it mediates the hepatic beneficial effects of GCGR agonist. Thus, it bears the therapeutic potential of hepatic steatosis.

## 2 Results

### 2.1 CD9 is Decreased in the Fatty Livers of Patients and Mice and Upregulates Upon GCGR Activation

**Figure 1:**
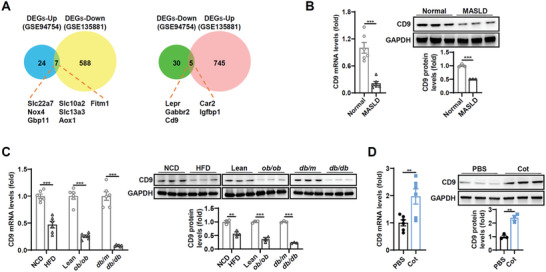
Hepatic CD9 is down‐regulated in the fatty livers of patients and mice, and increases upon GCGR activation. A) Venn diagrams of the differentially expressed genes (DEGs) from two mouse liver gene datasets, comparing the HFD‐induced hepatic steatosis (GSE94754) with fatty livers relieved by GCGR agonist treatment (GSE135881). B) The mRNA and protein levels of CD9 in the livers of patients with MASLD were determined by qPCR and western blot. C) The mRNA and protein levels of hepatic CD9 in the indicated mice. D) The mRNA and protein levels of CD9 in livers of HFD mice after treated with cotadutide acetate (Cot). n = 6 patients or mice per group for B–D. Data are presented as the mean ± S.E.M. Statistical analysis was performed using *t*‐test for B–D. ^**^, *p* < 0.01; ^***^, *p* < 0.001.

To screen for genes that may relate to GCGR agonist hepatic effects, we analyzed two publicly available gene expression datasets. One is liver from mice injected with a highly selective GCGR agonist (GSE135881) (Table S1, Supporting Information), which was reported to ameliorate hepatic steatosis.^[^ ^12^ ^]^ The other is HFD‐induced fatty livers (GSE94754) (Table S2, Supporting Information).^[^ ^20^ ^]^ As shown in **Figure** **1**A, 12 genes exhibited opposite expression changes between the GCGR agonist treatment and the disease model (*P* value of 0.01 and fold change greater than 1.5 as cutoff points), and among them, two genes of CD9 and NOX4 encode transmembrane proteins. Since GCGR is a transmembrane receptor, protein related to its transmembrane transduction always attracts attention.^[^ ^21^ ^]^ Indeed, CD9 and NOX4 expression were changed in livers after activation of GCGR signaling by Cot, and the alteration of CD9 was more pronounced than NOX4 (Figure S1A,B, Supporting Information). CD9, belonging to tetraspanins family, was previously revealed to initiate the processes of signal transduction in various cellular activities, such as cell adhesion, motility and senescence.^[^ ^22^, ^23^, ^24^ ^]^ We then investigated the possibility of CD9 involvement in hepatic effects of GCGR signaling. A marked reduction of CD9 at both mRNA and protein levels was observed in the livers of MASLD patients (Figure 1B). Consistently, CD9 expression was downregulated in the livers of mouse models (HFD, ob/ob and db/db) relative to the corresponding control (Figure 1C). Moreover, CD9 expression was induced in livers after activation of GCGR signaling by Cot in vivo and in vitro (Figure 1D; Figure S1C, Supporting Information).

### 2.2 CD9 Deletion in the Liver Aggravates Hepatic Steatosis Under HFD by Regulating Fatty Acid Metabolism

**Figure 2:**
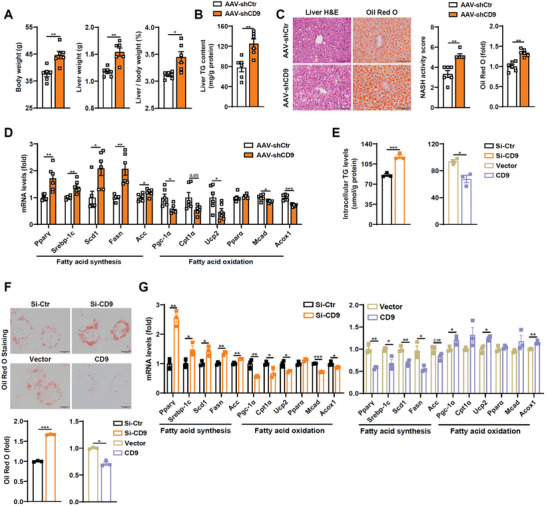
Hepatic CD9 deficiency exacerbates HFD‐induced hepatic steatosis. A–D) AAV‐shCtr and AAV‐shCD9 mice were fed on HFD for 12 weeks. (A) Body weight, liver weight and liver/body weight were determined. (B) Liver TG contents were determined enzymatically. (C) Representative H&E and Oil Red O staining of livers, and their quantifications. (D) The mRNA levels of genes related to lipid metabolism in livers. E–G) Huh7 cells were transfected with siRNA or plasmid for CD9 inhibition or overexpression, and then treated with free fatty acid (FFA) for 24 h. (E) The TG content was determined enzymatically. (F) Oil Red O staining and quantifications of treated Huh7 cells. (G) The mRNA levels of genes related to lipid metabolism were determined by qPCR. Scale bar = 100 µm for C, 10 µm for F. n = 6 mice per group for A–D, n = 3 per group for E–G. Data are presented as the mean ± S.E.M. Statistical analysis was performed using *t*‐test for A‐G. *, *p* < 0.05; ^**^, *p* < 0.01; ^***^, *p* < 0.001.

Next, we explored the impact of down‐regulated CD9 in the liver. Hepatic CD9 was knocked down with a recombinant adeno‐associated virus 8 vector (AAV8) containing the hepatocyte marker thyroxine‐binding globulin (TBG) promoter (i.e., AAV‐shCD9).^[^ ^25^ ^]^ AAV‐shCD9 treatment led to the CD9 specific deletion in livers, but not in skeletal muscle, adipose tissue, brain, or kidney of mice (Figure S2A,B, Supporting Information). We observed no significant differences between AAV‐shCD9 mice and AAV‐shCtr mice under normal chow diet (NCD), including body weight (BW), liver weight (LW), ratio of liver weight to body weight (LW/BW), blood glucose, serum and liver lipid contents and morphologic changes (Figure S2C‐H, Supporting Information). However, AAV‐shCD9 mice exhibited more aggravated hepatic steatosis in comparison with AAV‐shCtr mice with HFD challenging, showing increased BW, LW and LW/BW (**Figure** **2**A). We also obtained an elevated liver triglyceride (TG) content (Figure 2B), although hepatic cholesterol (TC) and serum biochemical indexes including blood glucose, glucose tolerance, TG, TC, LDL, and HDL were still unchanged (Figure S3A‐D, Supporting Information). Histology and Oil Red O staining further confirmed the pronouncedly exacerbated lipid accumulation in hepatocytes of AAV‐shCD9 mice (Figure 2C). Moreover, both RNA sequencing (RNA‐seq) and further qPCR analyses revealed that CD9 disruption primarily increased fatty acid synthesis genes, such as peroxisome proliferator‐activated receptor gamma (Pparγ), sterol regulatory element‐binding protein 1c (Srebp‐1c), stearoyl‐CoA desaturase 1 (Scd1), fatty acid synthase (Fasn) and acetyl‐CoA carboxylase (Acc), and decreased fatty acid oxidation genes of peroxisome proliferator‐activated receptor gamma coactivator 1‐alpha (Pgc‐1*α*), carnitine palmitoyltransferase 1 (Cpt1*α*), uncoupling protein 2 (Ucp2), medium‐chain acyl‐coenzyme A dehydrogenase (Mcad) and Acyl‐CoA oxidase (Acox1) (Figure 2D; Figure S3E, Supporting Information), rather than genes related to fatty acid uptake and transport (Figure S3F, Supporting Information). The increased fatty acid synthesis genes and decreased fatty acid oxidation genes were further validated by their protein expressions in livers of CD9 knockdown mice (Figure S3G, Supporting Information). In accordance with in vivo results, intracellular TG contents and lipid accumulation were enhanced in hepatocytes when transfected with CD9 small interfering RNA (siRNA), while attenuated with CD9 overexpression under free fatty acid (FFA) treatment (Figure 2E,F). Also, CD9 regulation of lipogenesis and lipolysis was further confirmed by related gene and protein expressions, as well as by ^13^C isotope assay evaluated de novo lipogenesis and fatty acid oxidation in vitro (Figure 2G; Figure S3H,I, Supporting Information). Taken together, these findings suggest that CD9 deficiency accelerates hepatic steatosis through enhancing fatty acid synthesis and suppressing oxidation under HFD.

### 2.3 CD9 Modulates Hepatic Fatty Acid Metabolism Through Regulating CFD Pathway via FLI1 Ubiquitination

**Figure 3:**
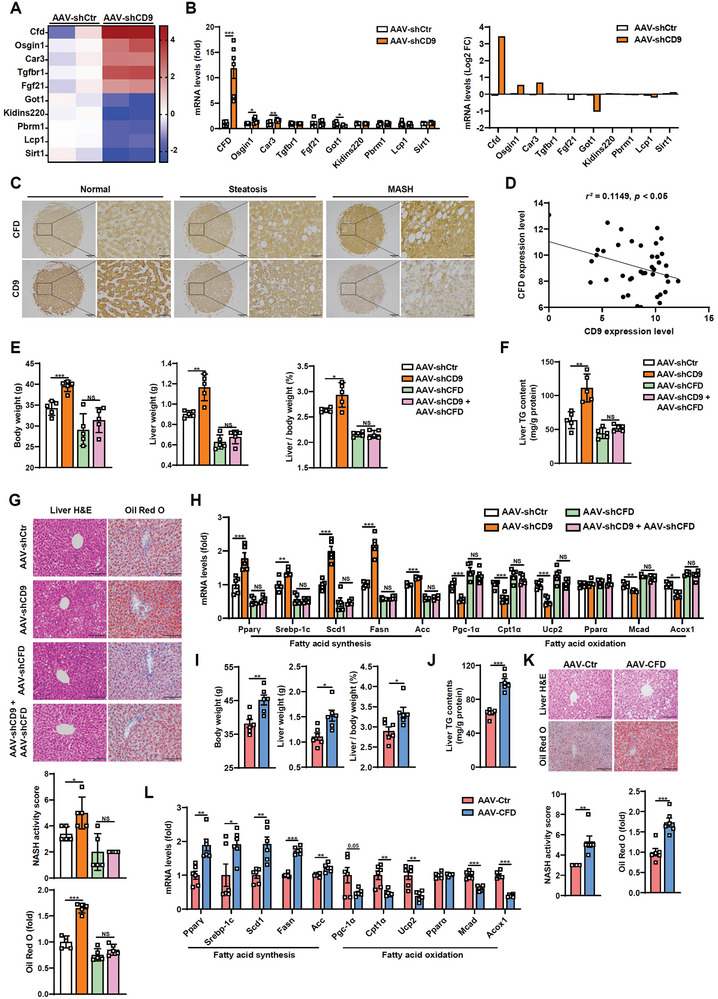
CD9 insufficiency promotes hepatic steatosis by inducing CFD expression. A) The heatmap displays the top 5 up‐regulated and down‐regulated differentially expressed genes in livers of HFD‐fed AAV‐shCtr and AAV‐shCD9 mice. B) The mRNA levels and their indicated log‐transformed fold change in livers of HFD‐fed AAV‐shCtr and AAV‐shCD9 mice. C) The IHC staining of paraffin‐embedded samples showed that the upregulated CFD and downregulated CD9 with MASLD progression. D) A scatter diagram showed negative correlation between CD9 and CFD in livers of MASLD patients (n = 40 samples). E–H) AAV‐shCtr and AAV‐shCD9 mice were injected with AAV8‐TBG‐shCFD and fed HFD for 10 weeks before sacrifice. (E) Body weight, liver weight and liver/body weight were determined. (F) Liver TG contents were determined enzymatically. (G) Representative H&E and Oil Red O staining of livers, and their quantifications. (H) The mRNA levels of genes related to lipid metabolism in livers. I–L) AAV‐Ctr and AAV‐CFD mice were fed on HFD for 12 weeks. (I) Body weight, liver weight and liver/body weight were determined. (J) Liver TG contents were determined enzymatically. (K) Representative H&E and Oil Red O staining of livers, and their quantifications. (L) The mRNA levels of genes related to lipid metabolism in livers. Scale bar = 200 and 50 µm for C, 100 µm for G and K. n = 5 mice per group for E‐H, n = 6 mice per group for B and I‐L. Data are presented as the mean ± S.E.M except mean for B (right panel). Statistical analysis was performed using *t*‐test for B, I‐L, Spearman's correlations for D, and one‐way ANOVA test for E‐H. ^*^, *p* < 0.05; ^**^, *p* < 0.01; ^***^, *p* < 0.001. Abbreviations: NS, not significant.

Then we explored the mechanisms underlying the hepatic effects of CD9. Heat map indicated the top five genes from the upregulated and downregulated differentially expressed genes (DEGs) in livers upon CD9 deletion through RNA‐seq analysis (**Figure** **3**A; Table S3, Supporting Information). By qPCR, the most one induced among the top‐ranked DEGs was identified, i.e., CFD, as showed a robust changed in mRNA expression (Figure 3B). Moreover, by analyzing human liver microarrays, we found the induction of CFD accompanying with the decline of CD9 during MASLD progression (Figure 3C), and a trend of negative correlation between them was displayed from the scatter plot (Figure 3D). Then, we clarified the possible involvement of CFD in CD9 effects. Knockdown of liver CFD by AAV8‐TBG‐shCFD significantly restrained CD9 ablation‐induced hepatic steatosis, including the alteration of BW, LW, LW/BW, TG content and fatty acid synthesis and oxidation related genes and proteins under HFD condition (Figure 3E‐H; Figure S4, Supporting Information). These results suggest that CD9 deletion accelerates hepatic steatosis mainly through CFD induction. Furthermore, we injected AAV8‐TBG‐CFD to overexpress CFD abundantly in the liver (Figure S5A,B, Supporting Information). A similar hepatic steatosis with CD9 ablated mice was observed, manifesting as increased BW, LW, LW/BW, liver TG content, lipid accumulation and fatty acid metabolic genes and proteins changed under HFD condition, although NCD‐fed CFD‐overexpressed mice appeared unchanged compared with controls (Figure 3I‐L; Figures S5C‐E and S6, Supporting Information). These data further prove the essential role of CFD in CD9 hepatic effects.

**Figure 4:**
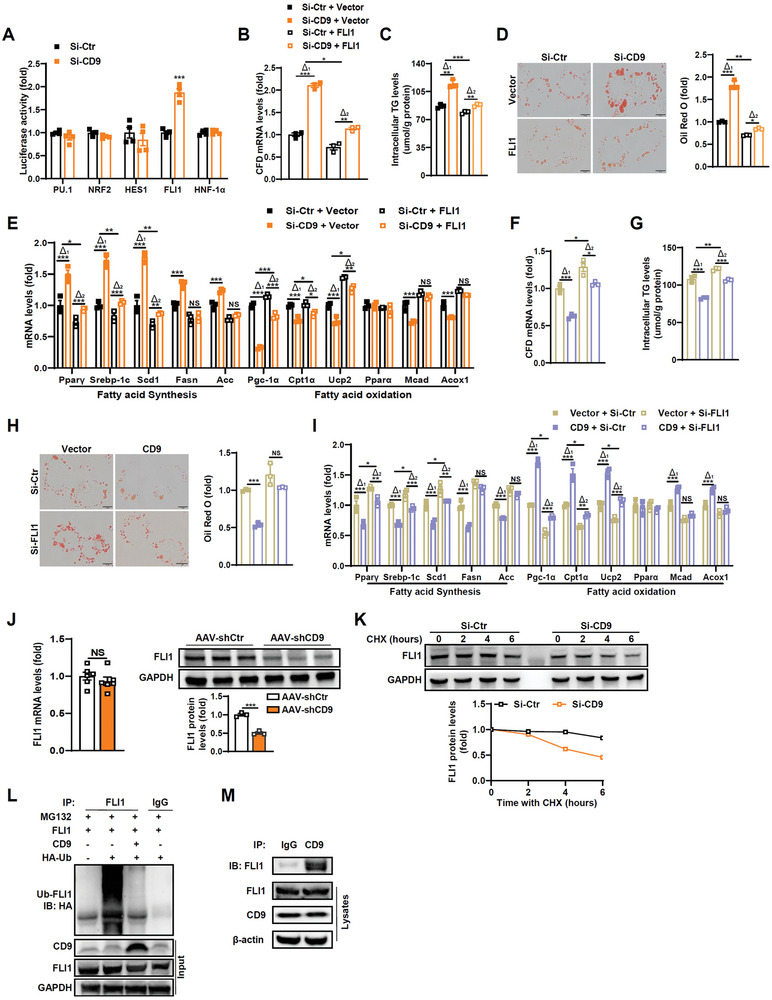
CD9 regulates CFD expression and hepatic fatty acid synthesis and oxidation genes through ubiquitination‐proteasomal degradation of FLI1. A) The luciferase reporter activities of indicated transcriptional factors were detected in Huh7 cells transfected by CD9‐specific siRNA with FFA treatment. B–E) Huh7 cells were co‐transfected with siRNA of CD9 and plasmid of FLI1, and then treated with FFA for 24 h. CFD mRNA level (B), TG content (C), Oil Red O staining with quantification (D) and the mRNA levels of genes related to lipid metabolism (E) were determined. F–I) Huh7 cells were co‐transfected with plasmid of CD9 and siRNA of FLI1, and then treated with FFA for 24 h. CFD mRNA level (F), TG content (G), Oil Red O staining with quantification (H) and the mRNA levels of genes related to lipid metabolism (I) were determined. J) The mRNA and protein levels of FLI1 in liver tissues of HFD‐fed AAV‐shCtr and AAV‐shCD9 mice. K) FFA‐treated Huh7 cells were transfected with CD9‐siRNA or control‐siRNA with cycloheximide (CHX) and harvested at different time points as indicated, protein expression and quantifications of FLI1 were assessed by immunoblotting. L) FFA‐treated Huh7 cells were transfected with indicated plasmids, the ubiquitination of FLI1 was assessed by immunoblotting. M) CD9 interacted with FLI1 by the coimmunoprecipitation assays in FFA‐treated Huh7 cells. Scale bar = 10 µm for D and H. n = 6 mice per group for J, n = 4 per group for A, n = 3 per group for B‐I. Data are presented as the mean ± S.E.M. Statistical analysis was performed using *t*‐test for A and J, and one‐way ANOVA test for B‐I. ^*^, *p* < 0.05; ^**^, *p* < 0.01; ^***^, *p* < 0.001. Abbreviations: NS, not significant.

We next addressed the molecular basis by which CD9 regulated CFD. The aforementioned in vivo results indicated that CFD mRNA was regulated by CD9 manipulation (Figure 3B), suggesting that CD9 modulated CFD might be the gene transcript level. Several transcription factors response elements binding sites have been identified in the promoter region of the CFD gene, such as binding sites for transcription factor PU.1,^[^ ^26^ ^]^ nuclear factor erythroid‐derived 2‐related factor 2 (NRF2),^[^ ^27^ ^]^ hairy and enhancer of split homologue‐1 (HES‐1),^[^ ^28^ ^]^ friend leukemia integration 1 (FLI1),^[^ ^29^ ^]^ hepatocyte nuclear factor‐1 alpha (HNF‐1*α*).^[^ ^30^ ^]^ Therefore, transcription factors were considered important for CFD transcript regulation. We then performed the transient transfection assay with above transcription factors, and the results showed CFD luciferase reporter gene activity altered in the presence of FLI1 but not by PU.1, NRF2, HES‐1 and HNF‐1*α* (**Figure** **4**A). To further confirm the interaction between FLI1 and CFD, chromatin immunoprecipitation assays were performed, revealing a PCR‐amplifiable sequence spanning from −990 to −795bp within the CFD promoter (Figure S7A,B, Supporting Information). We then assessed if FLI1 was responsible for CD9 effects in hepatocytes. The results showed modulation of FLI1 abated the effects induced by CD9‐siRNA or overexpression plasmid, including CFD expression, intracellular TG content, and fatty acid synthesis and oxidation related genes and proteins (Figure 4B‐I; Figure S8A,B, Supporting Information), indicating CD9 effects were mainly via FLI1. Moreover, the effects of CFD and FLI1 on hepatic lipid metabolism were further confirmed by ^13^C isotope assay evaluated de novo lipogenesis and fatty acid oxidation (Figure S8C, Supporting Information). To further address the mechanism of CD9 regulating FLI1, we measured FLI1 expression in AAV‐shCD9 mice, and found CD9 regulated FLI1 protein level but not mRNA expression (Figure 4J), which indicated a posttranslational regulation. Previous studies have shown that proteasomal degradation is likely to be the main mechanism of turnover for most E‐26 transformation‐specific (ETS) family members, including FLI1.^[^ ^31^, ^32^ ^]^ We then sought to determine if CD9 modulated FLI1 protein stability. Knockdown of CD9 significantly shortened, and overexpression of CD9 prolonged the half‐life of FLI1 protein (Figure 4K; Figure S8D, Supporting Information), and overexpressed CD9 decreased FLI1 ubiquitylation (Figure 4L). Furthermore, Lys‐48 (K48) and Lys‐63 (K63) were reported linked ubiquitin chains of FLI1,^[^ ^32^ ^]^ we then co‐expressed FLI1 with wild type HA‐ubiquitin or ubiquitin mutants that are deficient for Lys‐48‐ and Lys‐63‐linked chains (K48R and K63R). Immunoprecipitation of the ubiquitinated FLI1 revealed that CD9 overexpression decreased ubiquitination pattern of FLI1 was abrogated by both K48R and K63R HA‐ubiquitin, indicating that K48 and K63 are linked ubiquitin chain which mediated CD9 regulated FLI1 ubiquitylation (Figure S8E, Supporting Information). In addition, Gierisch et al. have screened lysine residue(s) that is responsible for the degradation of FLI1, and identified that Lys‐334 is the major site required for FLI1 protein ubiquitination.^[^ ^32^ ^]^ We therefore assessed whether this site mediated the effect of overexpressed CD9 on FLI1 ubiquitylation. By co‐expressing the mutant FLI1 (K344R) with HA‐ubiquitin, immunoprecipitation of the ubiquitinated FLI1 showed that CD9 overexpression inhibited FLI1 ubiquitination was abrogated by K344R mutant (Figure S8F, Supporting Information). Thus, K344 residue of FLI1 protein might be the key site for CD9 regulated FLI1 ubiquitination. To assess whether there is enzymatic activity associated with this process, we incubated hepatocytes with MG132 to inhibit protease activity, and found that the decreased FLI1 protein expression by CD9 downregulation was abrogated (Figure S8G, Supporting Information). CD9 was also found its interaction with FLI1 by coimmunoprecipitation assay (Figure 4M). To further assess the subcellular localization of the CD9‐FLI1 interaction, we co‐stained CD9 with the plasma membrane marker sodium potassium ATPase (NKA). The results revealed that the subcellular location of CD9 is at the plasma membrane. And the co‐staining of CD9 with FLI1 was showed their colocalization, indicating the CD9‐FLI1 interaction is near the plasma membrane (Figure S8H, Supporting Information). Taken together, these data indicate CD9 regulates CFD by ubiquitination‐proteasomal degradation of FLI1.

### 2.4 CD9 Influences Body Weight by Modulating Lipogenesis and Thermogenesis of Adipose Tissue Through CFD

**Figure 5:**
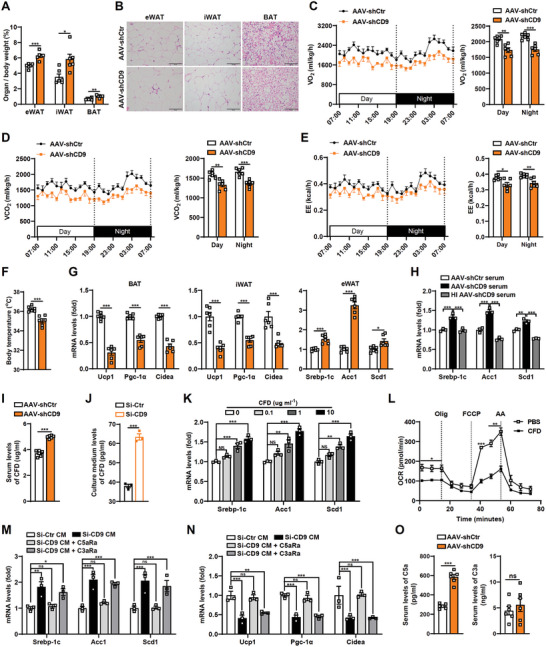
CD9 affects body weight by controlling lipogenesis and thermogenesis of adipose tissue through CFD. A‐G) AAV‐shCtr and AAV‐shCD9 mice were fed on HFD for 12 weeks. (A) The ratio of 3 adipose tissues (eWAT, iWAT, BAT) to body weight were determined. (B) Representative H&E staining of eWAT, iWAT and BAT. (C‐E) Oxygen consumption rate (C), carbon dioxide production rate (D) and whole‐body energy expenditure (E) were monitored for a 24‐h recording period and the average quantification for 12‐h day and 12‐h night. (F) Body temperature. (G) The mRNA levels of genes related to fatty acid synthesis and thermogenesis in adipose tissues. H) Expression of fatty acid synthesis genes in differentiated 3T3‐L1 adipocytes incubated with medium containing 10% (v/v) serum or heat‐inactivated (HI) serum from the indicated groups of mice. I) Serum level of CFD in HFD‐fed AAV‐shCtr and AAV‐shCD9 mice were measured by ELISA. J) Cultured Huh7 cells were transfected with siRNA of CD9 or control, respectively, and then treated with FFA for 24 h. CFD concentration in cell culture supernatant was measured. K) Differentiated 3T3‐L1 adipocytes were treated with CFD of concentrations of 0.1, 1, 10 ug/ml, and the mRNA levels of fatty acid synthesis genes were determined. L) Oxygen consumption rate (OCR) in fully differentiated stromal‐vascular fraction (SVF) cells incubated with or without recombinant CFD. M,N) Differentiated 3T3‐L1 adipocytes (M) or SVF cells (N) were treated with the culture medium of FFA‐treated mouse primary hepatocyte with CD9 knockdown, along with antagonists of C3aR or C5aR, and the mRNA levels of lipogenesis and thermogenesis genes were assessed, respectively. O) Serum level of C3a and C5a in HFD‐fed AAV‐shCtr and AAV‐shCD9 mice were measured by ELISA. Scale bar = 100 µm for B. n = 6 mice per group for A‐G, I and O, n = 3 per group for H and J‐N. Data are presented as the mean ± S.E.M. Statistical analysis was performed using *t*‐test for A, C‐E, F, G, I, J, O, one‐way or two‐way ANOVA test for H, K‐N. ^*^, *p* < 0.05; ^**^, *p* < 0.01; ^***^, *p* < 0.001. Abbreviations: NS, not significant.

In addition, having observed an interesting alteration of body weight after CD9 deletion in the liver (Figure 2A), we next examined the potential mechanism. In order to determine the cause of body weight change, we weighed adipose tissues, including epididymal white adipose tissue (eWAT), inguinal WAT (iWAT) and brown adipose tissue (BAT). All three adipose tissues of AAV‐shCD9 mice exhibited higher weight and enlarged adipocyte size in comparison to AAV‐shCtr mice (**Figure** **5**A,B). Body weight and adiposity are balanced by food intake and energy expenditure. Indirect calorimetry study indicated CD9 influences energy expenditure, including VO_2_ consumption, VCO_2_ production, energy expenditure (EE) and body temperature (Figure 5C‐F), but not food intake, physical activity and respiratory exchange ratio (Figure S9A‐C, Supporting Information). In line with above results, we assessed decreased thermogenic genes including uncoupling protein 1 (Ucp1), Pgc‐1*α* and cell death–inducing DFFA‐like effector a (Cidea) in BAT and iWAT, as well as elevated lipogenesis genes in eWAT of CD9 deficient mice (Figure 5G). These data imply that changes of body weight after CD9 deletion in the liver may be attributed to enhanced lipogenesis in eWAT and impaired thermogenesis in iWAT and BAT. We therefore speculated that the CD9‐deficient liver could possibly communicate with adipose tissues through circulation. To test this hypothesis, serum from AAV‐shCD9 mice was applied to 3T3‐L1 adipocytes, and the results showed that AAV‐shCD9 serum substantially induced the lipogenesis genes; while heat‐inactivated (HI) AAV‐shCD9 serum completely abolished its stimulatory effect (Figure 5H), indicating the possible protein existing in the circulation of liver CD9‐deficient mice that regulates the function of adipocytes. CFD was previously reported as a circulating factor,^[^ ^33^ ^]^ and then we measured the serum levels of CFD. Serum CFD levels were induced in AAV‐shCD9 mice (Figure 5I), and consistently, CFD levels were higher in the cell culture supernatants of CD9‐siRNA hepatocytes (Figure 5J). In addition, the expressions of CFD in adipose tissues (eWAT, iWAT, and BAT) were unchanged after CD9 knocking down (Figure S9D, Supporting Information). We applied CFD recombinant protein to 3T3‐L1 adipocytes and stromal‐vascular fraction (SVF) cells isolated from the brown fat tissue respectively, and found dose‐dependently elevated lipogenesis genes (Figure 5K), as well as a markedly inhibited uncoupled O_2_ consumption rate (Figure 5L). These data indicate the cross‐talk between hepatic CD9 and adipose tissues may also associate with CFD. To further confirm this possibility, antagonists of two CFD receptors, which were reported mainly mediated CFD controlled lipid metabolism in adipocyte,^[^ ^34^, ^35^, ^36^ ^]^ i.e., C3aR and C5aR were adopted. Blocking C5aR, but not C3aR abrogated the altered lipogenesis and thermogenesis genes of adipocytes when incubated with conditioned medium from hepatocytes transfected with CD9‐siRNA (Figure 5M,N), indicating CFD is in charge of hepatic CD9 regulated adipocyte function, which mainly via adipocyte C5aR. This was further confirmed by elevated serum levels of C5a in hepatic CD9‐deficient mice rather than C3a (Figure 5O).

### 2.5 Rescuing CD9 in the Liver Alleviates Hepatic Steatosis

**Figure 6:**
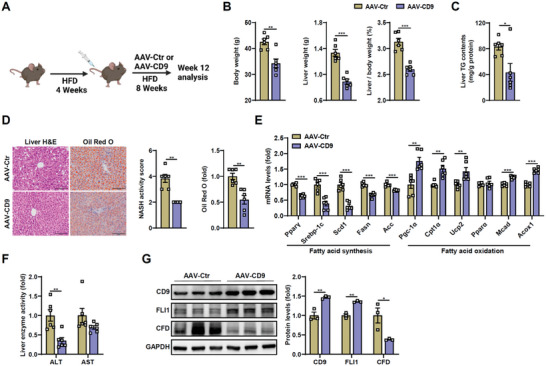
CD9 overexpression in the liver ameliorates HFD‐induced hepatic steatosis. AAV‐Ctr and AAV‐CD9 mice were fed on HFD for 12 weeks. A) Schematic diagrams illustrating the experimental design and treatment groups. B) Body weight, liver weight and liver/body weight were determined. C) Liver TG contents were determined enzymatically. D) Representative H&E and Oil Red O staining of livers, and their quantification. E) The mRNA levels of genes related to lipid metabolism in livers. F) Serum levels of liver enzyme activity (ALT and AST) were determined enzymatically. G) Western blot and its quantification of CD9, FLI1 and CFD in liver tissues. Scale bar = 100 µm for C. n = 6 mice per group for B‐G. Data are presented as the mean ± S.E.M. Statistical analysis was performed using *t*‐test for B‐G. ^*^, *p* < 0.05; ^**^, *p* < 0.01; ^***^, *p* < 0.001.

Next, we evaluated the effects of rescuing hepatic CD9 expression in HFD mice (**Figure** **6**A). Liver CD9 overexpression by AAV8‐TBG‐CD9 significantly reduced BW, LW and LW/BW (Figure 6B), and improved hepatic TG, lipid accumulation and related lipid gene and protein expressions (Figure 6C‐E; Figure S10, Supporting Information). Interestingly, the liver enzyme activity of alanine aminotransferase was significantly decreased, indicating liver function was also improved by AAV‐CD9 (Figure 6F). Moreover, CD9 reinforcement decreased CFD and increased FLI1 protein levels (Figure 6G), which was consistent with our aforementioned mechanism observations. These findings indicate that rescuing of CD9 mitigates MASLD development.

### 2.6 CD9 Medicates Hepatic Benefits of GCGR Activation

**Figure 7:**
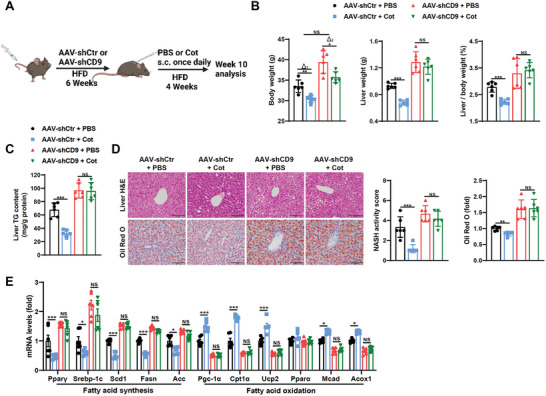
Deletion of CD9 abolishes the hepatic beneficial effects of cotadutide in HFD mice. AAV‐shCtr and AAV‐shCD9 mice were fed on HFD for 10 weeks, and received equimolar dosing (30 nmol kg^−1^ (body weight)) subcutaneously, once daily for 4 weeks at week 6 of HFD feeding. A) Schematic diagrams illustrating the experimental design and treatment groups. B) Body weight, liver weight and liver/body weight were determined. C) Liver TG contents were determined enzymatically. D) Representative H&E and Oil Red O staining of livers, and their quantification. E) The mRNA levels of genes related to lipid metabolism in livers. Scale bar = 100 µm for C. n = 6 mice per group for B–E. Data are presented as the mean ± S.E.M. Statistical analysis was performed using one‐way ANOVA test for B‐E. ^*^, *p* < 0.05; ^**^, *p* < 0.01; ^***^, *p* < 0.001. Abbreviations: NS, not significant.

Finally, we checked whether CD9 was involved in the hepatic effects of the GCGR agonist (**Figure** **7**A). A GCGR/GLP1R dual agonist Cot was utilized as it had been proved the liver effects were predominantly through GCGR, and extrahepatic improvement via GLP1R.^[^ ^11^ ^]^ Indeed, Cot alleviated liver steatosis, showing as reduced BW, LW, LW/BW, liver TG content and morphology, as well as altered fatty acid synthesis and oxidation genes and proteins, which was in accordance with recent report.^[^ ^11^ ^]^ Delating hepatic CD9 by AAV‐TBG‐shCD9 significantly abolished the remission of steatosis and lipid accumulation by Cot treatment, except BW (Figure 7B‐E; Figure S11, Supporting Information). These results indicate CD9 is responsible for the hepatic beneficial effects of Cot, which is considered to be attributed to GCGR signaling, but not extrahepatic weight improvement attributes to GLP1R.^[^ ^11^ ^]^

## 3 Discussion

MASLD, characterized by aberrant TG accumulation in the liver, influences the metabolic remodeling of hepatic and peripheral tissues, and leads to fibrosis, cirrhosis, liver failure and cancer. However, there is still no effective curative treatment. Amounting evidence is providing for molecule agonists targeting endocrine peptide receptor, such as dual or triple agonist peptides at GCGR, GLP1R, and GIPR to be developed into clinical promising anti‐MASLD treatment candidates.^[^ ^8^, ^9^, ^10^ ^]^ Recent studies indicated GLP1R and GIPR agonism offer improved effects on food intake, weight loss and glycemic control, whereas action on the liver to reduce lipid content is primarily mediated through GCGR agonism.^[^ ^11^, ^12^, ^13^ ^]^ Despite demonstrating marked phenotypic improvements, the mechanisms of GCGR agonist on liver remain incompletely understood. Based on our present study, we uncover an essential role of CD9 in counteracting hepatic steatosis, and highlight it as a mediator of the hepatic effects of GCGR agonist.

Our study clarified a vital role of CD9 in hepatic lipid accumulation and steatosis. Analysis of fatty livers from both humans and mice provided clinical and basic pathological insights into the association of decreased CD9 levels with hepatic steatosis, a correlation that was functionally validated by liver CD9 knockdown in vivo and in vitro. CD9 was described to associate with cancer prognosis,^[^ ^37^, ^38^ ^]^ and participated in glomerular disease progression,^[^ ^39^ ^]^ atherosclerotic plaque formation^[^ ^24^ ^]^ and entry of SARS‐CoV‐2.^[^ ^40^ ^]^ Here we demonstrated that the function of CD9 in regulating liver TG content through influencing fatty acid synthesis and oxidation. Notably, lipid accumulation induced by CD9 ablation was only seen with HFD feeding, but not under basal conditions. This phenomenon has also been observed in several pathogenic genes related to MASLD, such as transmembrane 6 superfamily member 2,^[^ ^41^ ^]^ as well as mitochondrial glycerol 3‐phosphate dehydrogenase which was reported by our previous study.^[^ ^42^ ^]^ Moreover, we found hepatic CD9 inhibition could also impair systemic metabolism and promote fat adipose deposition and obesity via enhancing lipogenesis and weakening thermogenesis of adipose tissue, which indicated the involvement of CD9 in the functional integration between the liver and adipose tissue.

Our results identified the responsibility of CFD in mediating hepatic CD9 effects. Liver‐specific CFD deletion abrogated CD9 effects, and overexpressed CFD induced consistent hepatic steatosis observed in CD9 knockdown mice. Our human tissue microarray analysis further provided clinic association of increased CFD and reduced CD9 expression during the pathologic progression of MASLD. Another study on patients with alcohol‐associated hepatitis also proposed CFD was positively associated with disease severity, and might be a risk factor for mortality for patients.^[^ ^43^ ^]^ In addition, CFD was also responsible to hepatic CD9‐deficient induced obesity, which was in accordance with previous clinical human studies that implicated increased serum CFD levels in obesity^[^ ^44^, ^45^, ^46^ ^]^ and a positive correlation with body mass index.^[^ ^45^ ^]^ Moreover, our study suggests that CD9 regulated CFD is due to FLI1 proteasomal degradation. FLI1 belongs to the ETS transcription factor family, whose member are strong activators or repressors of transcription with a highly conserved ETS domain.^[^ ^47^ ^]^ Considering their high conservation, proteasomal degradation is likely to be the main mechanism of turnover for most ETS family members, including FLI1.^[^ ^31^, ^32^ ^]^ Besides, FLI1's transcriptional regulation of CFD was also reported in endothelial cells by previous study, that it contributed to the onset of pulmonary arterial hypertension in systemic sclerosis.^[^ ^29^ ^]^

We uncovered CD9 was responsible for the hepatic benefits of GCGR agonist. Cot, a GCGR/GLP1R agonist, caused the reduction of blood glycaemia, body weight and hepatic steatosis in patients with T2DM.^[^ ^14^ ^]^ Previous study indicated its action to reduce liver lipid content is predominantly through GCGR agonism, and the improvement of body weight and glucose control are mediated through GLP1 signaling.^[^ ^11^ ^]^ A recent randomized phase 2a trial of Cot provides evidence of additional benefits in human livers could be attributed to GCGR engagement.^[^ ^15^ ^]^ Our results suggest CD9 does not bear the responsibility of GlP1R's action on weight loss, but contributes to GCGR signaling on the remission of liver steatosis. Moreover, our findings propose that CD9 might serve as a potential therapeutic target for MASLD, which was evidenced by that the restoration of CD9 attenuated lipid accumulation and hepatic steatosis. Tetraspanins are always considered as useful therapeutic targets due to the transmembrane structure that function in mediating diverse biological processes,^[^ ^48^ ^]^ such as injury repair and infectious diseases.^[^ ^16^, ^18^ ^]^ Moreover, the impact of CD9 on liver steatosis and its mediation in GCGR agonist were investigated in this study. Nevertheless, the possibility of the involvement of NOX4 in GCGR function cannot be conclusively dismissed, and warrants further investigation in the future.

Take together, our study reveals the essential role of CD9 in counteracting liver steatosis. Specifically, CD9 alters genes related to hepatic fatty acid synthesis and oxidation through regulating CFD expression via ubiquitination‐proteasomal degradation of FLI1. CD9 also affects body weight by controlling lipogenesis and thermogenesis of adipose tissue. Additionally, our study highlights CD9 as a mediator of GCGR agonist's hepatic benefits, and may server as a promising therapeutic target for liver steatosis.

## 4 Experimental Section

### Bioinformatic Analysis

Two datasets for hepatic steatosis were included, i.e., GSE94754 and GSE135881, from the National Center for Biotechnology Information (NCBI) Gene Expression Omnibus (GEO). Then, by setting *P* value less than 0.01 and fold change greater than 1.5 or less than 0.67 as cutoff points, it undertook intersection analysis of the two groups of differentially expressed genes. The Venn diagrams of DEGs were shown in Figure 1A.

### Animals

All studies involving mouse experiments were approved by the Laboratory Animal Welfare and Ethics Committee of the Army Military Medical University (AMUWEC20201372). C57BL/6J, ob/ob, db/m and db/db male mice were purchased from GemPharmatech Co., Ltd. All mice were housed at room temperature with a standard 12‐h light/dark cycle with ad libitum access to food and water. Body weight was assessed weekly. Body temperature was monitored by using a rectal probe. To clarify the difference caused by diet, mice were divided into normal chow diet (NCD) and high fat diet (HFD, fat, 60 Kcal%; protein, 20 Kcal%; carbohydrates, 20 Kcal%; Research Diets, New Brunswick, NJ) from 7‐week‐old randomly. Age‐matched littermates served as controls which were randomly divided into groups, and the groups did not present differences in body weights before the treatments. All investigators were blind to the allocation of groups when conducting histology and pathology analyses. Male mice were used in the study and were treated as described in the Figure legends, and the number of mice used for the study was decided according to previous reports. Exclusion criteria were based on animal well‐being before the experiments started.

### Human Samples

The experimental protocols were approved by the Ethics Committee of Xinqiao Hospital, and consistent with the Declaration of Helsinki (Institutional Review Board‐approved protocol number 2016‐056‐01). All samples were collected from patients hospitalized at Chongqing Medical University, and written consent was obtained from each participant. The procedure was carried out in a manner similar to that in our previous study.^[^ ^42^ ^]^ Briefly, liver samples were obtained from individuals with MASLD who underwent percutaneous liver biopsy, and control samples were collected from liver transplant donors. The diagnostic criteria for MASLD were as follows: i) performed a physical examination, laboratory investigation, and liver biopsy. ii) other causes of liver disease were ruled out, such as current or past excessive alcohol consumption (defined as average daily consumption of alcohol >20 g for males or >10 g for females), chronic hepatitis C or B, autoimmune, celiac disease, genetic disorders based on self‐reports, or if laboratory and/or histopathological data showed causes of liver disease other than MASLD. All liver specimens were snap frozen after resection and stored at −80 °C.

### Cotadutide Study in HFD C57BL/6J

To understand the effects with cotadutide treatment, a study utilizing HFD‐10‐week male mice was performed. Animals received a daily subcutaneous injection with PBS or cotadutide acetate (30 nmol kg^−1^ (body weight)) 2 h prior to lights out for an additional 4 weeks. Cotadutide acetate was obtained from Med Chem Express (HY‐P2231A).

### Adeno‐Associated Virus (AAV)‐Mediated Gene Knockdown and Overexpression

An adeno‐associated virus delivery system was used to specifically knock down or overexpress murine gene in mouse hepatocyte. The AAV shRNA vector pAAV‐TBG‐GdGreen‐miR30shRNA‐WPRE and AAV cloning vector pAAV‐TBG‐3xFlag‐p2a‐GdGreen‐WPRE were obtained from Obio Technology (Shanghai). To achieve hepatocyte‐specific CD9‐deficient mice (AAV‐shCD9), CFD‐deficient mice (AAV‐shCFD), CD9‐overexpression mice (AAV‐CD9) and CFD‐overexpression mice (AAV‐CFD), mice were transduced with AAV serotype 8 vectors (2 × 1011 v.g. per mouse, via the tail vein), and AAV8‐TBG‐GFP served as control (AAV‐shCtr/AAV‐Ctr).

### Glucose Tolerance Test (GTT)

Before the tests, mice were fasted for 16 h, and then injected intraperitoneally with a solution of D‐glucose (2 g kg^−1^ body weight, Sigma). Tail blood glucose levels were measured at different time points post‐injection using the Accu‐Chek Performa and test strips (Roche).

### Metabolites Measurement

Liver and serum samples were collected from the indicated mice after overnight fasting. Liver (TG and TC) and serum (TG, TC, HDL, and LDL) metabolites levels were measured by corresponding commercial determination kits (Nanjing Jiancheng) according to the manufacturer's instructions.

### Hematoxylin & Eosin (H&E), Immunohistochemistry (IHC), Immunofluorescence (IF) and Oil Red O Staining

H&E, IHC, IF and Oil Red O staining were carried out as was previously described.^[^ ^42^ ^]^ Briefly, paraffin sections were prepared for H&E staining and IHC (CD9, SA35‐08 from Novus; CFD, orb156818 from Biorbyt) following standard procedures. For Oil Red O staining, sections and cells were fixed in formaldehyde solution and stained with Modified Oil Red O Staining Kit (C0158M, Beyotime). For immunofluorescence analysis, cells were prepared for IF (CD9, SA35‐08 from Novus, CFD, orb156818 from Biorbyt, sodium potassium ATPase, ab76020 from Abcam, FLI1, GTX112937 from GeneTex, DAPI, C1005 from Beyotime) and observed with a confocal laser scanning microscope (ZEISS).

### Cell Culture and Treatment

All cells were grown in a humidified atmosphere containing 5% CO_2_ at 37 °C. Huh7 cells were purchased from Procell Life Science&Technology Co., Ltd. 3T3‐L1 cells were purchased from the Cell Bank of the Chinese Academy of Sciences. All cell identities were confirmed and cultured as recommended by the supplier. The cellular lipid accumulation model was induced by free fatty acid (FFA, including palmitate and oleic acid at a final ratio of 1:2), which was added to the medium for 24 h. For knockdown, cells were seeded in 6‐well culture plates and transiently transfected with 30 pmol siRNA oligonucleotides with 5 µL RNAiMAX (Life Technology) per well. CD9‐specific (Si‐CD9), FLI1‐specific (Si‐FLI1), CFD‐specific (Si‐CFD) and corresponding negative control (Si‐Ctr) siRNAs were synthesized by Sangon Biotech. For intracellular gene overexpression, cells were transfected with plasmids at 1 µg per well with Lipofectamine3000 Reagent (Invitrogen) after cells attachment. The CD9, FLI1, HA‐Ub and control vector plasmids were generated by Sino Biological. MG132 was purchased from Santa Cruz Biotechnology.

### RNA Isolation and Gene Expression Analysis

RNA was isolated from cultured cells or tissue samples using RNAiso Plus (Takara) according to the manufacturer's instructions. RNA quality was assessed on a Nanodrop2000, where samples with a 260/280 ratio of 1.8–2.0 were processed for subsequent gene analyses. 1000 ng of mRNA was reverse‐transcribed into cDNA using the PrimeScript RT Reagent Kit with gDNA Eraser (Takara) according to the manufacturer's protocol. TB Green (Takara) was applied to quantify PCR amplification. The primer pairs used in this study are described in Table S4 (Supporting Information).

### Chromatin Immunoprecipitation (ChIP)‐qPCR Analysis

ChIP analysis was performed with the commercial kit (Abcam, ab500) according to its protocols. Huh7 were collected and fixed in formaldehyde at room temperature for 10 min. The chromatin was sonicated into optimal fragments with lysis buffer, and then incubated overnight with control IgG or anti‐FLI1 antibodies (GeneTex) at 4 °C. Protein A sepharose beads were used for antibody pulldown. Then the ChIP'd DNA was washed, reverse cross‐linked, and purified following the kit protocols. PCR primer sequences for ChIP‐qPCR are listed in the Table S4 (Supporting Information).

### RNA‐seq Preparation and Sequencing

Total RNA was harvested from the livers of HFD AAV‐shCtr and AAV‐shCD9 mice with RNAiso Plus (Takara) and quantified by a NanoDrop2000. RNA integrity and gDNA contamination were tested by denaturing agarose gel electrophoresis, and the sequencing library was determined by an Agilent 2100 Bioanalyzer using the Agilent DNA 1000 Chip Kit (Agilent). RNA‐seq analyses were performed by KangChen Biotech.Shanghai, using the Illumina HiSeq 4000 platform.

### Stable Isotope‐Labeled Fatty Acid De Novo Synthesis and Oxidation Analysis

For lipid de novo synthesis, hepatocytes were incubated in fresh medium for 6 h, and then ^13^C labeling glucose were added in medium 12 h before sampled. Fatty acid de novo synthesis was evaluated according to the mass of ^13^C labeling glucose‐derived ^13^C into the fatty acid palmitate acid, which was assessed by ultra‐performance liquid chromatography and mass spectrometry (UPLC‐MS/MS) analysis. For ^13^C labeling fatty acid oxidation assessment, hepatocytes were treated by ^13^C labeling palmitic acid for 12 h, which undergoes degradation to form ^13^C_2_‐acetyl‐CoA. Subsequently, this acetyl‐CoA combines with oxaloacetate in the tricarboxylic acid cycle to generate ^13^C_2_‐citrate (the M+2 isotopologue). The mass of M+2 isotopologue citrate was assessed by UPLC‐MS/MS, which was performed by Bioegene and Biotree Co., Ltd. (Shanghai, China).

### Human Liver Tissue Microarray Chips

Human liver tissue microarray chips were purchased from Bioaitech (D170Lv01) and Taibosi Biological Technology Co., Ltd (DLV851, DLV1601, and DLV20812a); for scanned images of H&E staining, corresponding clinical information and ethical materials please refer to: https://www.bioaitech.com/chip‐design and https://www.taibsbio.com). Histological classification was performed in a blinded fashion by an experienced gastrointestinal pathologist with the NAFLD activity score system (NAS).^[^ ^49^, ^50^ ^]^ Briefly, the scoring system comprised three of which were evaluated semi‐quantitatively: steatosis (evaluation of parenchymal involvement: <5%, 5–33%, 33–66%, >66%, scored 0–3), lobular inflammation (assessment of inflammatory foci per 200× field: no foci, <2 foci, 2–4 foci, >4 foci, scored 0–3), and hepatocellular ballooning (none, few balloon cells, many cells/prominent ballooning, scored 0–2). Individual scores were assigned for each parameter. NAS of ≥5 correlated with a diagnosis of MASH, and histology with scores of less than 3 were diagnosed as steatosis.^[^ ^51^ ^]^ A total of 71 samples, including 31 normal liver tissues, 19 steatosis and 21 MASH were analyzed. The immunohistochemistry tissue microarray stained slides were scored as described previously,^[^ ^52^ ^]^ considering i) the percentage of positive cells (P) was grouped into five categories, i.e., 0 (<5%), 1 (5–25%), 2 (25–50%), 3 (50–75%), 4 (75–100%), and ii) staining intensity (I) was grouped into four categories (color from light to dark), i.e., 0, 1, 2, 3. The final score was calculated as P x I and correlations between CD9 and CFD expression were analyzed by Pearson correlation coefficient. *p* < 0.05 was considered to indicate significant difference.

### Luciferase Assay

The promoter region of CFD was cloned into the MCS‐firefly luciferase reporter vector. Huh7 cells were seeded in 24 well plates and transfected with Si‐Ctr or Si‐CD9. Twenty‐four hours later, cells were grown to 70–80% confluency and co‐transfected with the CFD reporter plasmid and Renilla luciferase plasmid, along with PU.1, NRF2, HES1, HNF1*α*, and FLI1 expression plasmids. Then, firefly and Renilla luciferase activities were determined by the Dual‐Luciferase Reporter System (Promega) according to the manufacturer's instructions. The firefly luciferase activity was normalized to Renilla luciferase activity.

### Western Blots

For protein extraction, cells were lysed in sample buffer (50 mm Tris‐HCl, pH 6.8, 2% SDS, 10% glycerol, 100 mm dithiothreitol, and 0.1% bromophenol blue), and tissue lysates were prepared as previously described.^[^ ^53^ ^]^ Protein concentrations were measured using the BCA Protein Assay Kit (Beyotime). Extracted protein lysates were resolved by SDS‐PAGE and immunoblotted with the indicated primary antibodies (1:500–1:1000) and their corresponding HRP‐conjugated secondary antibodies. Blots were developed with chemiluminescent HRP substrate (Millipore) and imaged using a fusion FX5s system (Vilber Lourmat). The anti‐CD9 (SA35‐08) and anti‐PGC1*α* (NBP1‐04676) were obtained from Novus. The anti‐CFD was obtained from R&D (AF5430). The anti‐FLI1 was obtained from GeneTex (GTX112937). The anti‐NOX4 (14347‐1‐AP), anti‐ACC1 (21923‐1‐AP), anti‐ACOX1 (10957‐1‐AP), anti‐CPT1a (15184‐1‐AP), anti‐PPARγ(16643‐1‐AP), anti‐PPAR*α* (66826‐1‐lg), anti‐SCD1 (28678‐1‐AP), anti‐FASN (10624‐2‐AP) and anti‐UCP2 (11081‐1‐AP) were obtained from Proteintech. The anti‐SREBP‐1 was obtained from Santa Cruz (sc‐365513). The anti‐ACADM was obtained from Abcam (ab92461). Anti‐GAPDH antibody was obtained from Aksomics (Shanghai).

### Ubiquitination, Half‐Life Assays and Immunoprecipitation

Ubiquitination and half‐life assays were performed according to our previous protocol.^[^ ^42^ ^]^ Briefly, for the ubiquitination assay, cells were transfected by FLI1 plasmid with hemagglutinin (HA)‐tagged ubiquitin, with or without CD9 plasmid for 24 h and then treated with FFA and MG‐132 (10 µm). Cells were harvested, and the lysates were incubated with anti‐FLI1 antibody at 4 °C overnight. Immunoprecipitated proteins were analyzed by immunoblotting with an antibody directed against the HA epitope. To measure FLI1 protein half‐life, cells were treated with Si‐CD9 and FFA, and then cycloheximide (CHX, 25 µm) was added to block protein synthesis. Total lysates were collected at the indicated time points after CHX administration and subjected to immunoblot analysis. For immunoprecipitation, cell lysates were collected at 48 h post‐transfection in RIPA buffer containing 10 mm sodium phosphate (pH 8.0), 150 mm NaCl, 1% Triton X‐100, 1% sodium deoxycholate, and 0.1% SDS, with the addition of 1 mm dithiothreitol, 1 mm phenylmethylsulfonyl fluoride (PMSF), and protease inhibitor cocktail (PIC). Cell lysates were incubated with 1 µg of antibody with 10 µL of protein A‐agarose beads on a rotator at 4 °C overnight. The immunoprecipitated complexes were washed and eluted in sample buffer by boiling for 5 min. The samples were then resolved by SDS‐PAGE and transferred onto nitrocellulose membranes for immunoblotting analysis. Primary antibodies were used as described in the Western blot part.

### Metabolic Cage Measurement

Oxygen consumption rate (VO_2_, ml/kg/h), carbon dioxide output (VCO_2_, ml/kg/h) and physical activity were measured at the end of treatment using the Comprehensive Lab Animal Monitoring System (CLAMS, Columbus Instruments) according to the manufacturer's instructions. Mice were allowed to acclimatize for 24 h prior to collection of data. Voluntary activity was monitored from *x* axis beam breaks collected every 18 min. Respiratory exchange ratio (RER) values were calculated as VCO_2_/VO_2_. Quantity of heat (kcal/h) was calculated from gas exchange.

### ELISA

CFD, C3a and C5a concentrations in mouse serum and cellular media were measured respectively by commercial ELISA kit (Abnova KA3822), (D721062, Sangon Biotech) and (ab193718, Abcam) according to the manufacturer's instructions.

### 3T3‐L1 Cells Differentiation

Cells were seeded in 6‐well plates and cultured in 2.5 mL DMEM (containing 10% fetal bovine serum, FBS) and were grown to confluency. Two days after confluency, cells were changed to induction medium (DMEM containing 10% FBS, 1 µm dexamethasone, 5 µg mL^−1^ insulin and 0.5 mm isobutylmethylxanthine) for 2 days. Cells were maintained in maintenance medium (DMEM containing 10% FBS and 5 µg mL^−1^ insulin) for another 4 days. Fresh maintenance medium was replaced every 2 days. Induced adipocytes were washed twice with DMEM and cultured in DMEM for another 6 h. Recombinant protein CFD (R&D) or culture medium of FFA‐treated mouse primary hepatocytes with CD9 knockdown was added to fully differentiated 3T3‐L1 adipocytes for an additional 2 days before analysis. PBS buffer, C3aR1 antagonist‐calbiochem (C3aRa, SB 290157, Millipore), and C5aR1 antagonist‐PMX 205 Trifluoroacetate (C5aRa, Med Chem Express) were applied in subsequent tests.

### Primary Mouse Pre‐Adipocytes Culture and Differentiation

The interscapular brown fat pad was isolated from newborn mice, and then minced and digested for 30 min in warm‐up isolation buffer (containing 0.75 mg mL^−1^ type II collagenase, Sigma). The cell suspension was filtered with a 100 µm cell strainer, then centrifuged for 10 min at 1500 rpm to pellet the SVF cells.^[^ ^54^ ^]^ For pre‐adipocyte differentiation, SVF cells were seeded in poly‐L‐lysine coated 6‐well plates with DMEM (containing 10% FBS) and grown to confluence. Then confluent cells were changed to induction medium (DMEM containing 10% FBS, 1 µm dexamethasone, 1 µg mL^−1^ insulin, 0.5 mm isobutylmethylxanthine, 10 µm pioglitazone and 1 nm triiodothyronine) for 2 days. Cells were maintained in maintenance medium (DMEM containing 10% FBS, 5 µg mL^−1^ insulin and 1 nm triiodothyronine) for another 4 days. Fresh maintenance medium was replaced every 2 days. Subsequent processing was similar to the3T3‐L1 differentiation protocol described above.

### Cellular Respiration Assay

The O_2_ consumption rate (OCR) of differentiated primary adipocytes was determined using an Agilent Seahorse XF extracellular flux analyzer (Agilen) according to our previous description.^[^ ^55^ ^]^ After differentiation, adipocytes were maintained at 60–80% confluence in XF cell culture plates, and the real‐time change in OCR was then monitored following sequential injection of 1 mM oligomycin A, 1 mm carbonyl cyanide 4‐(trifluoromethoxy) phenylhydrazone (FCCP), and 0.5 mm rotenone. Data were calculated from three independent measurements obtained prior to or after compound injection.

### Statistics and Reproducibility

Quantitative values were presented as the mean ± S.E.M unless otherwise indicated. The values for n represent biological replicates for cell experiments. For animal experiments, n corresponds to the number of animals per condition. Specific details for n values were noted in each figure legend. Statistical analyses were performed by GraphPad Prism 8. Differences between the two experimental groups were analyzed by Student *t*‐test. For multiple comparison analyses, one‐way or two‐way ANOVA was used. When overall *F* tests were significant (*p* < 0.05), Tukey post hoc testing was conducted for adjustment. For OCR experiment, two‐way repeated‐measures ANOVA with Tukey post hoc testing was used. Significant differences between the two groups (^*^ *p* < 0.05, ^**^ *p* < 0.01, ^***^ *p* < 0.001) were defined.

## Conflict of Interest

The authors declare no conflict of interest.

## Author Contributions

Y.Z., Y.W., X.X., L.Z., and J.Z. contributed equally to this work. Y.Z., Y.W., X.X., and L.Z. designed and conducted in vivo and in vitro experiments, acquisition of data, and performed statistical analysis. H.Q., X.L., Y.W., and B.H. performed analysis and interpretation of data. J.Z., Y.W., and X.X. performed animal studies and helped with data analysis. Y.Z. and X.X. performed drafting of the manuscript. Y.Z., H.Z., J.L., G.Y. and Z.Z. performed critical revision of the manuscript for important intellectual content. H.Z., Y.Z., and H.Q were the guarantor of this work and, as such, had full access to all the data in the study and took responsibility for the integrity of the data and the accuracy of the data analysis.

## Supporting information

Supporting Information

Supporting Information

## Data Availability

The data that support the findings of this study are available on request from the corresponding author. The data are not publicly available due to privacy or ethical restrictions.
