# Supplementary material for: CD9 Counteracts Liver Steatosis and Mediates GCGR Agonist Hepatic Effects
Source: Adv Sci (Weinh). 2024 Jun 5;11(29):2400819. doi: 10.1002/advs.202400819 (PMC11304330; doi:10.1002/advs.202400819)
Supplement: Supplementary file 1 — Supporting Information [file ADVS-11-2400819-s001.pdf]

ADVANCED SCIENCE
Open Access

Supporting Information

for *Adv. Sci.*, DOI 10.1002/advs.202400819

CD9 Counteracts Liver Steatosis and Mediates GCGR Agonist Hepatic Effects

Yi Zheng, Yuren Wang, Xin Xiong, Linlin Zhang, Jiaran Zhu, Bangliang Huang, Xiufei Liu, Jinbo Liu, Zhiming Zhu, Gangyi Yang, Hua Qu* and Hongting Zheng**

CD9 counteracts liver steatosis and mediates GCGR agonist hepatic effects

Supplementary Information

Figure S1. CD9 and NOX4 expressions responding to cotadutide treatment.

Figure S2. AAV-shCD9 mice show similar body weight and tissue morphology under NCD.

Figure S3. Blood glucose, liver TC, serum lipid contents and lipid metabolism in AAV-shCD9 mice under HFD conditions.

Figure S4. The protein levels of fatty acid synthesis and oxidation genes in HFD-fed AAV-shCD9 mice after CFD knockdown.

Figure S5. Body weight and liver phenotype are comparable in AAV-CFD mice as compared with AAV-Ctr mice under NCD.

Figure S6. The protein levels of fatty acid synthesis and oxidation genes in HFD-fed AAV-CFD mice.

Figure S7. Sequence information of CFD promoter and chromatin immunoprecipitation assay showing the binding of FLI1 on CFD promoter.

Figure S8. CD9 regulates hepatic fatty acid synthesis and oxidation through ubiquitination-proteasomal degradation of FLI1.

Figure S9. Hepatic CD9 deficiency in livers does not affect physical activity, respiratory exchange ratio and adipose tissue of CFD expression under HFD.

Figure S10. The protein levels of fatty acid synthesis and oxidation genes in HFD-fed AAV-CD9 mice.

Figure S11. The protein levels of fatty acid synthesis and oxidation genes in HFD-fed AAV-shCD9 mice after treated with cotadutide.

Table S1. RNA sequencing analysis of mice treated with a highly selective GCGR agonist (quoted from GEO#GSE135881).

Table S2. RNA sequencing analysis of mice with HFD-induced fatty livers (quoted from GEO#GSE94754).

Table S3. Top 5 upregulated and downregulated genes in livers upon CD9 deletion through RNA sequencing analysis.

Table S4. List of primers.

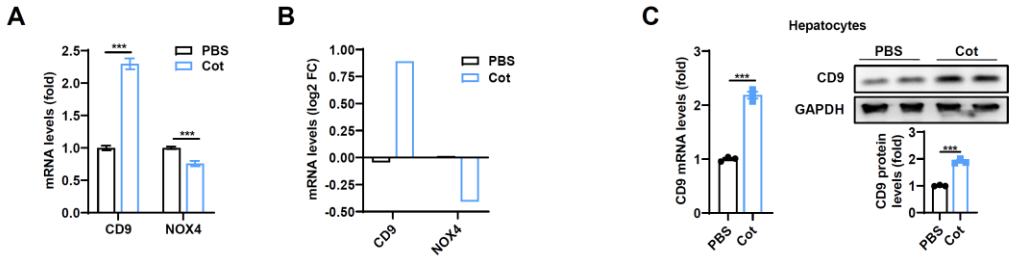

Figure S1. CD9 and NOX4 expressions responding to cotadutide treatment.

(A and B) The mRNA levels (A) and their indicated log-transformed fold change (B) of CD9 and NOX4 in livers of HFD mice treated with cotadutide acetate (Cot). (C) Huh7 cells were treated by free fatty acid (FFA) for 24h, the mRNA and protein levels of CD9 were assessed after Cot treatment.

n = 6 mice per group for A and B, n = 3 for C. Data are presented as the mean ± S.E.M. ***, $P < 0.001$.

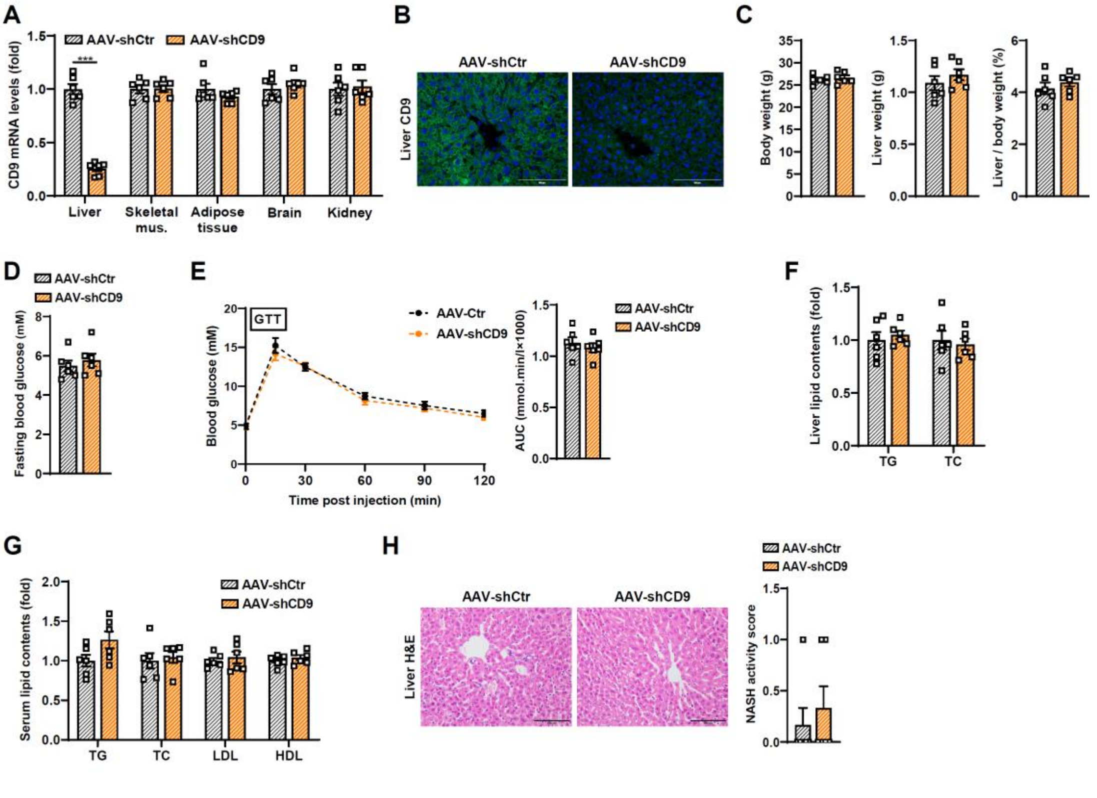

Figure S2. AAV-shCD9 mice show similar body weight and tissue morphology under NCD.

(A-H) AAV-shCtr and AAV-shCD9 mice were fed on NCD for 12 weeks. (A) AAV-shCD9 leads to CD9 attenuation exclusively in the liver. (B) Immunofluorescence analyses validates the suppression of CD9 in mouse liver with AAV-shCD9 treatment. (C) Body weight, liver weight and liver/body weight were determined. (D and E) At week 12 of NCD feeding, fasting blood glucose (D) and glucose tolerance test (GTT, E) were performed after overnight fasting. (F) Liver TG and TC were determined enzymatically. (G) Serum levels of TG, TC, LDL and HDL were

determined enzymatically. (H) H&E staining and NASH activity score of livers.
Scale bar = 100 μm for B and H. n = 6 mice per group for A-H. Data are presented as the mean ± S.E.M. ***, *P* < 0.001.

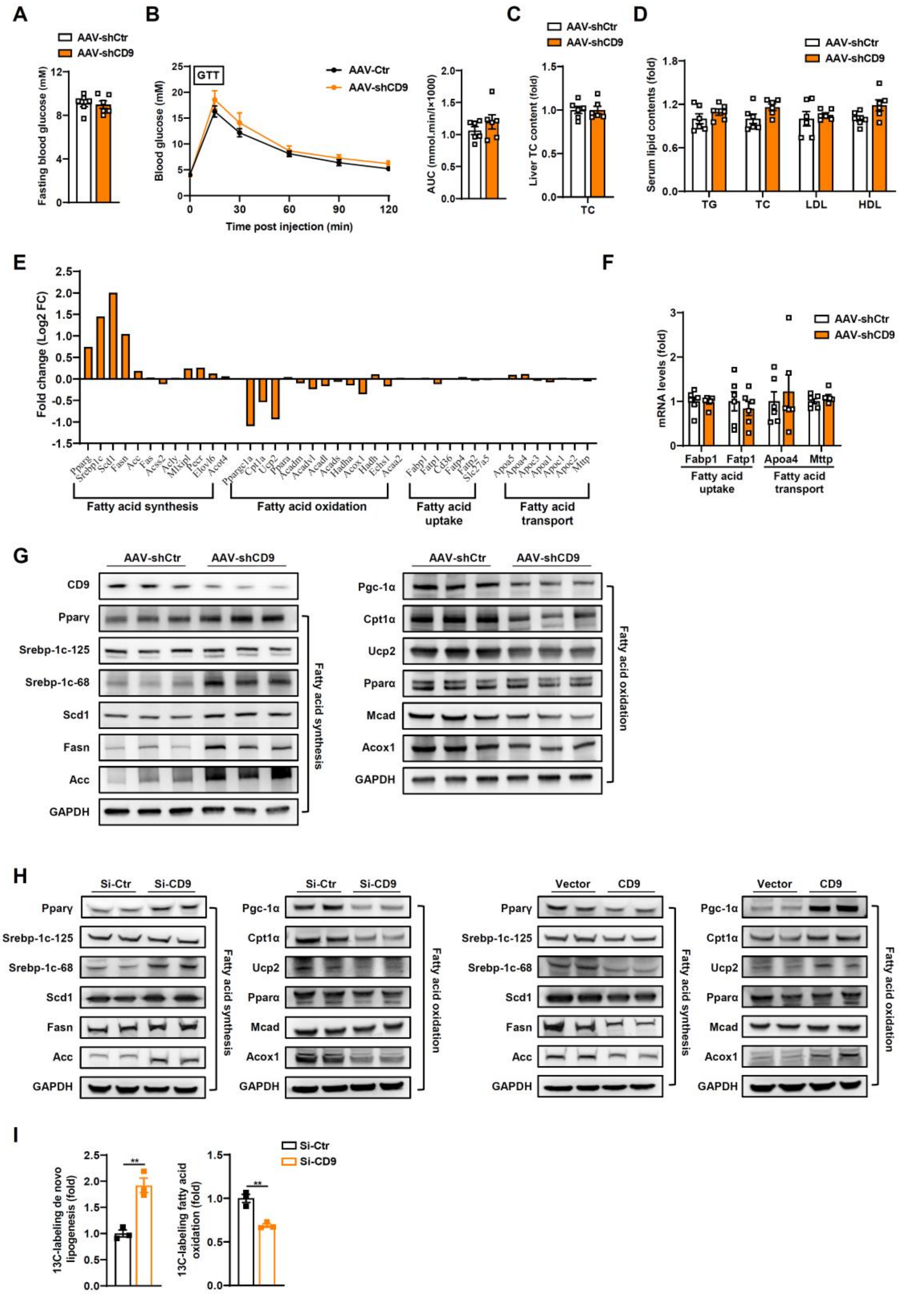

Figure S3. Blood glucose, liver TC, serum lipid contents and lipid metabolism in AAV-shCD9 mice under HFD conditions.

(A and B) At week 12 of HFD feeding, fasting blood glucose (A) and glucose tolerance test (GTT, B) was performed after overnight fasting. (C) Liver TC was determined enzymatically. (D) Serum levels of TG, TC, LDL and HDL were determined enzymatically. (E) RNA sequencing analysis of lipid metabolic pathway. (F) The mRNA levels of genes related to lipid uptake and transport in livers. (G) Protein levels of CD9 and genes related to fatty acid synthesis and oxidation. (H and I) Huh7 cells were transfected with siRNA or plasmid for CD9 inhibition or overexpression, and then treated with FFA for 24 hours. Protein levels of genes related to fatty acid synthesis and oxidation (H) and 13C labeling assay evaluated de novo lipogenesis and fatty acid oxidation (I) were shown.

n = 6 mice per group for A-D, F and G, n = 3 for H and I. Data are presented as the mean ± S.E.M. **, $P < 0.01$.

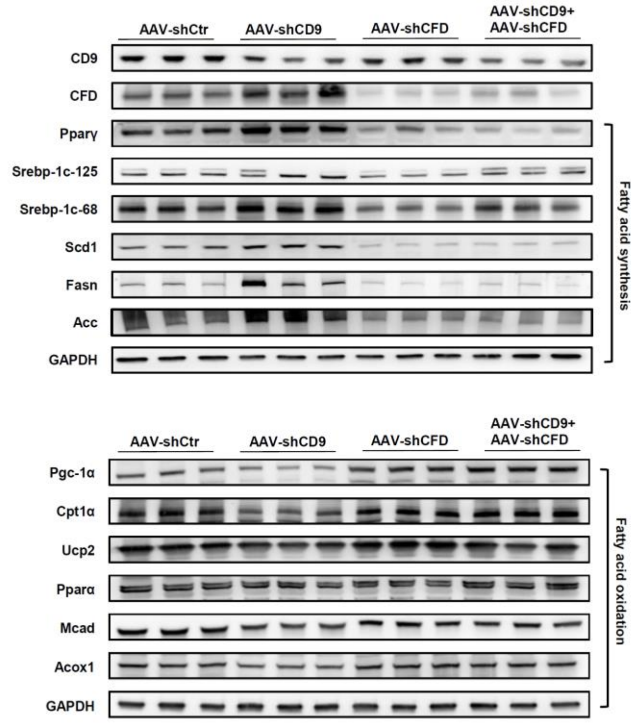

Figure S4. The protein levels of fatty acid synthesis and oxidation genes in HFD-fed AAV-shCD9 mice after CFD knockdown.

AAV-shCtr and AAV-shCD9 mice were injected with AAV8-TBG-shCFD and fed HFD for 10 weeks before sacrificed. The protein levels of CD9, CFD, and genes related to fatty acid synthesis and oxidation in livers were assessed. n = 5 mice per group.

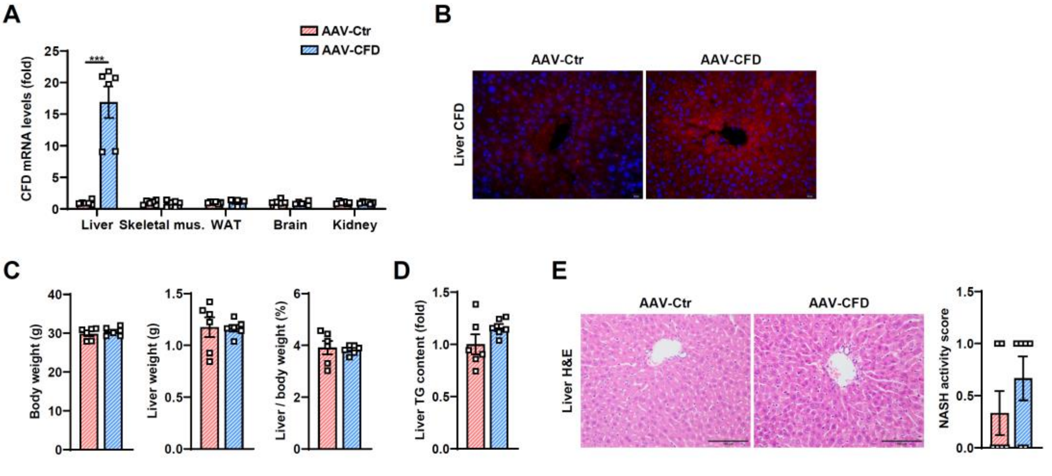

Figure S5. Body weight and liver phenotype are comparable in AAV-CFD mice as compared with AAV-Ctr mice under NCD.

(A-E) AAV-Ctr and AAV-CFD mice were fed on NCD for 12 weeks. (A) AAV-CFD leads to CFD overexpression exclusively in the liver. (B) Immunofluorescence analysis validates the overexpression of CFD in mouse liver with AAV-CFD treatment. (C) Body weight, liver weight and liver/body weight were determined. (D) Liver TG was determined enzymatically. (E) H&E staining and NASH activity score of livers.

Scale bar = 20 μm for B, 100 μm for E. n = 6 mice per group for A-E. Data are presented as the mean ± S.E.M. ***, $P < 0.001$.

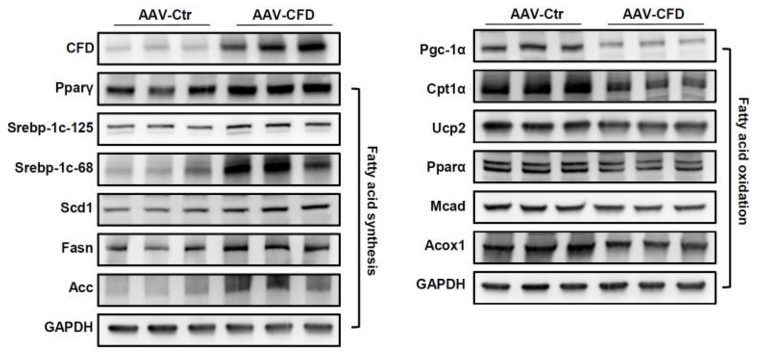

Figure S6. The protein levels of fatty acid synthesis and oxidation genes in HFD-fed AAV-CFD mice.

AAV-Ctr and AAV-CFD mice were fed on HFD for 12 weeks before sacrificed. The protein levels of CFD and genes related to fatty acid synthesis and oxidation in livers were assessed. n = 6 mice per group.

A

TAATTTTTTTGTAGAGATGGGGTCTCGCCGTCTTCCCCAAGCTGGCCTTGAATTCCTGGGCTCAAGAGATGCTCCTATC……….79
TGGGTCTCTCAATGTGCCGGGATGGTAGGTGGGAGCCACTGTGCCCGGCCAGCCCTGACTCTGTTCATCAGAACCTG……..156
CCCTCAGTGAACAGTTTGGGAGGGGGCGTGTGATCCAGGCTCCACCAACCCAAGGCACCCTTGAGACTTGGCTGGA……...232
GTGAGGAAGAATTTTAGGAGGGGCCCACTGAGCCACTGGGTTGAGTGTGGGGTCCCTGGAAACACACCAGGGGAGG……..308
CTGGTGAGGAAAGAAGCCATCAGAGAAGAAAGGAGAAGCCAGAAGCAGACACTGGACATTGTTCAGGCACCTGGATC……..385
AAGCCACACCTGAAGGCGGACATCCCTGAACTTTCCATATAACTTTCATGATTCAGGCTCCACCCAGTTTGTCTGGGTT……...464
TTCCTGTCCCTTGTAGCCAGTCTTCCCAGCTGCCTCTGCCTTGGCCTTTCCAACTTTCTATTCGTCAGGGTGTCAAGCC……..543
CCAGAGGCCACAGGGACAGAGACAAAGGTGTCAAGTGACCTTCCTACCCCCAACAGACCTTCTCTCTAATATAGAGGA……..621
GGAAAAGAAGGGAGGGTAGCAGGACAAGAAAGTGGGTTGTTTACACGCTGTGGCCTCCAGCTAGGATTGTGAAACAC……..698
CCTGGGGGACTCTGGGTGTGGTGAAGTTGGGGCCGGGGTGGGTGCAGCCCCCCTCTCTGCCTCCTGCCCATCTGA………773
GGGACTGCCCCCCACCAGGGCCAGCAGGCTGTCAGGCTCACAGAGGCCCGAGGGTCTCCCAGCGACTTGTCGACT……...848
CTGGTGTGAGTCTGGCAGGAGGTAACCCAGTCCCGGGGAGGGCGGAGGCTGGGCGGGCGAGTTCCCCTTTCCCCA……...923
GCTGGGCCGCTGCCCACCCGGGGAGCGGCCCGCCCCCTCCACCCTCATAAAAGCACCCCCAGGGCCCTGCCTGGG……..998
TCAGTGTCTCAGCCACAGCGGCTTCACC

B

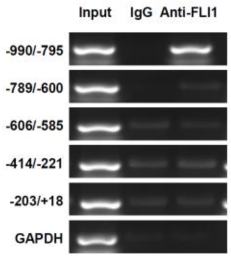

Figure S7. Sequence information of CFD promoter and chromatin immunoprecipitation assay showing the binding of FLI1 on CFD promoter.

(A and B) Sequence information of CFD promoter (A) and chromatin immunoprecipitation assay using different lengths of CFD promoter fragments (B) were shown.

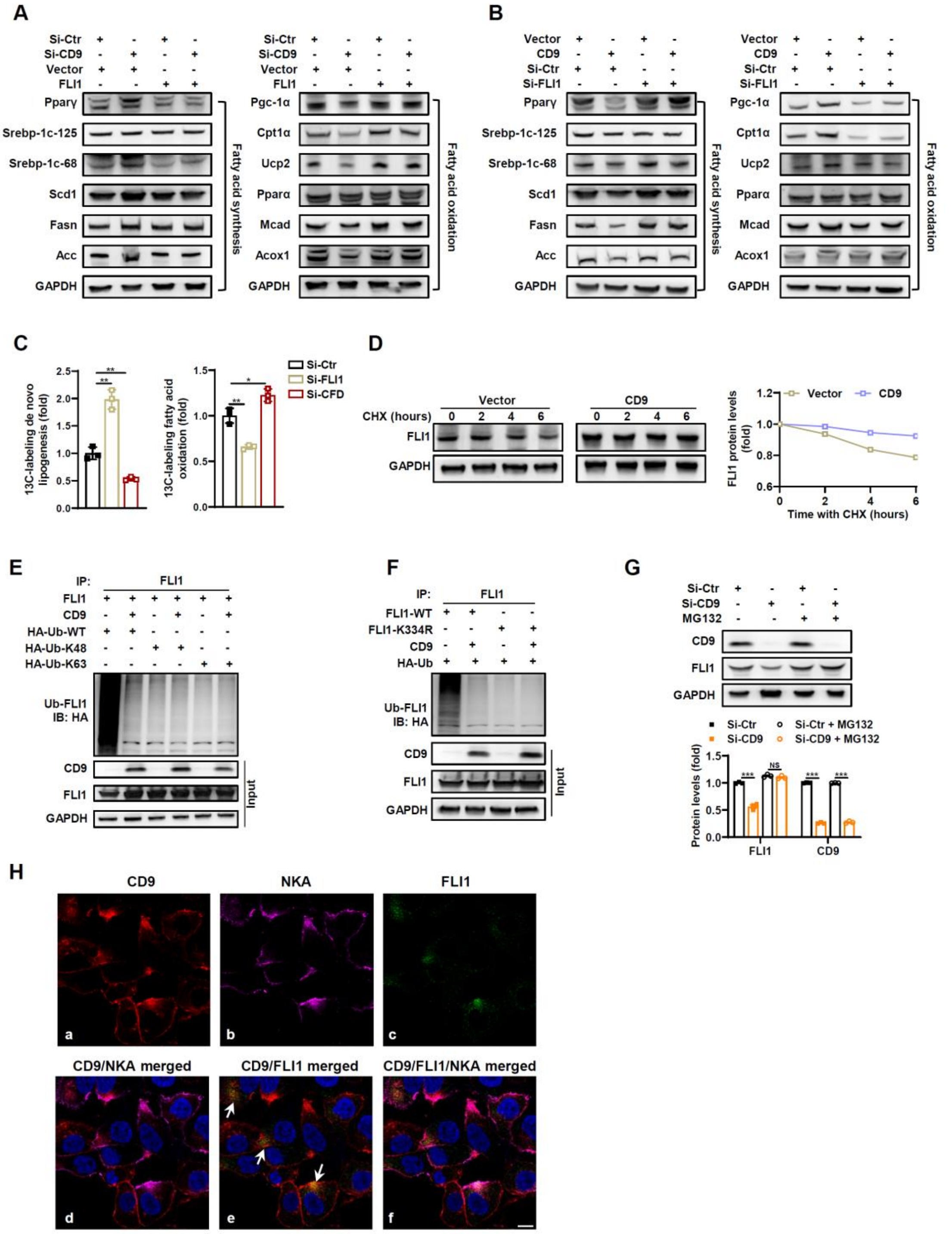

Figure S8. CD9 regulates hepatic fatty acid synthesis and oxidation through ubiquitination-proteasomal degradation of FLI1.

(A) Huh7 cells were co-transfected with siRNA of CD9 and plasmid of FLI1, and then treated with FFA for 24 hours, the protein levels of genes related to lipid metabolism were determined. (B) Huh7 cells were co-transfected with plasmid of CD9 and siRNA of FLI1, and then treated with FFA for 24 hours, the protein levels of genes related to lipid metabolism were determined. (C) FFA treated Huh7 cells were labeled by ^{13}C isotope, de novo lipogenesis and fatty acid oxidation were assessed under CFD or FLI1 manipulated conditions. (D) FFA-treated Huh7 cells

were transfected with CD9 overexpressed plasmids or control-vector with cycloheximide (CHX) and harvested at different time points as indicated, protein expression of FLI1 were assessed by immunoblotting. (E) FFA-treated Huh7 cells were co-expressed FLI1 with wild type (WT) HA-ubiquitin or ubiquitin mutants (K48R and K63R), immunoprecipitation of the ubiquitinated FLI1 were assessed. (F) FFA-treated Huh7 cells were transfected with indicated WT and mutant plasmids of FLI1, the ubiquitination of FLI1 was assessed by immunoblotting. (G) FFA-treated Huh7 cells were transfected with CD9-siRNA or control-siRNA with or without MG132 to inhibit protease activity, protein expression of FLI1 were assessed by immunoblotting. (H) FFA-treated Huh7 cells were co-stained of CD9, FLI1 and the plasma membrane marker sodium potassium ATPase (NKA), the subcellular location was shown.

Scale bar = 10 µm for H. White arrow indicates the overlapping of CD9 and FLI1. n = 3 for A-C, and G. Data are presented as the mean ± S.E.M. *, P < 0.05, **, P < 0.01. ***, P < 0.001. Abbreviations: NS, not significant.

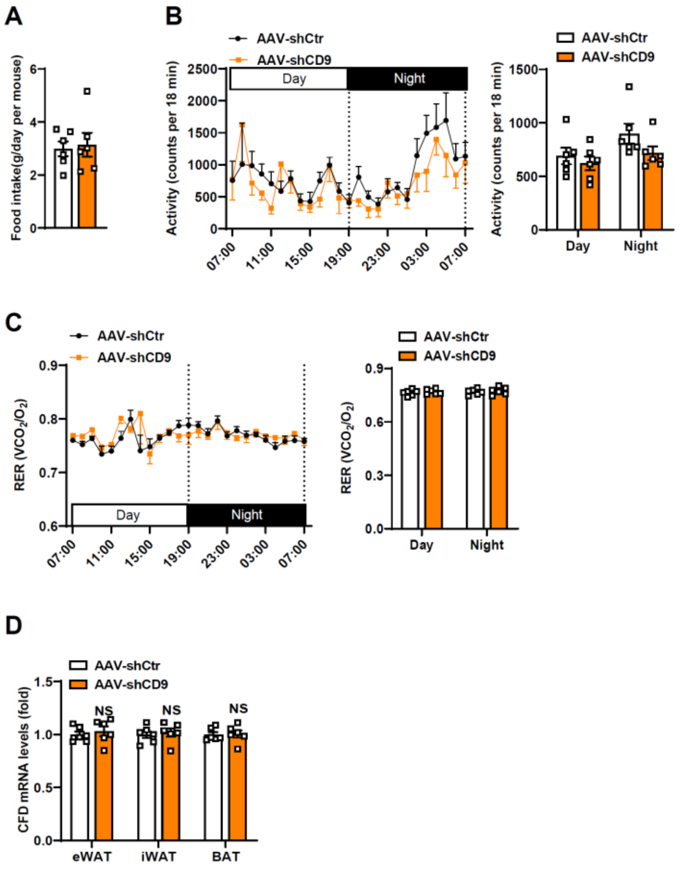

Figure S9. Hepatic CD9 deficiency in livers does not affect physical activity, respiratory exchange ratio and adipose tissue of CFD expression under HFD.

AAV-shCtr and AAV-shCD9 mice were fed on HFD for 12 weeks. (A) Food intake was determined. (B and C) Physical activity (B) and respiratory exchange ratio (RER, C) for a 24 h recording period

and their quantifications. (D) The mRNA expression of CFD were assessed in 3 adipose tissues (eWAT, iWAT, BAT).

n = 6 mice per group for A-D. Data are presented as the mean ± S.E.M. Abbreviations: NS, not significant.

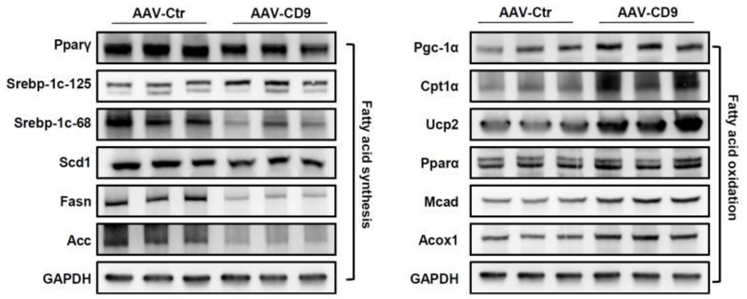

Figure S10. The protein levels of fatty acid synthesis and oxidation genes in HFD-fed AAV-CD9 mice.

AAV-Ctr and AAV-CD9 mice were fed on HFD for 12 weeks. The protein levels of genes related to lipid metabolism in livers were shown.

n = 6 mice per group.

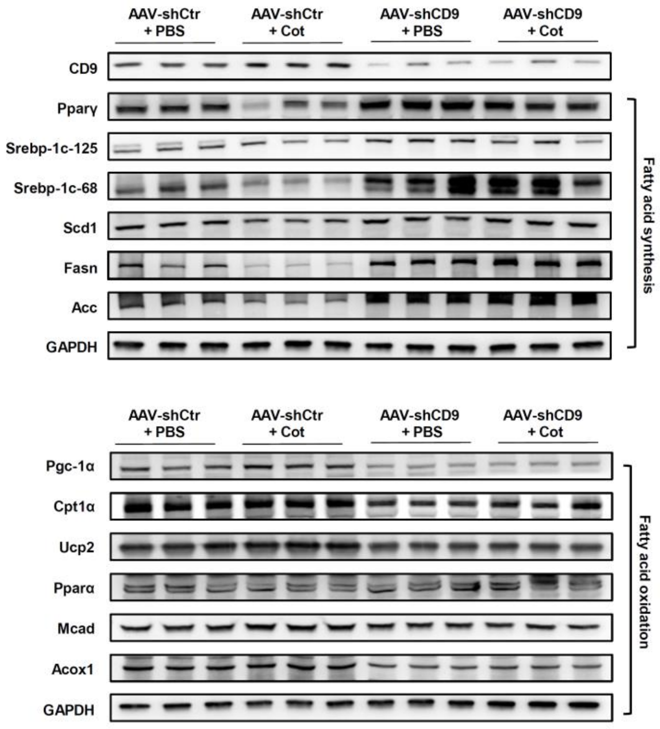

Figure S11. The protein levels of fatty acid synthesis and oxidation genes in HFD-fed AAV-shCD9 mice after treated with cotadutide.

AAV-shCtr and AAV-shCD9 mice were fed on HFD for 10 weeks, and received equimolar dosing (30 nmol per kg (body weight)) subcutaneously, once daily for 4 weeks at week 6 of HFD feeding. The protein levels of CD9 and genes related to lipid metabolism in livers.
n = 6 mice per group.
