# Supplementary material for: CD9 Counteracts Liver Steatosis and Mediates GCGR Agonist Hepatic Effects
Source: Adv Sci (Weinh). 2024 Jun 5;11(29):2400819. doi: 10.1002/advs.202400819 (PMC11304330; doi:10.1002/advs.202400819)
Supplement: Supplementary file 2 — Supporting Information [file ADVS-11-2400819-s002.zip › advs202400819-sup-0002-cif/Table S4.docx]

**Table S4. List of primers**

| Primers (H for human, m for mouse) | | |
| --- | --- | --- |
| m-CD9 | Forward | ATCAAATACCTGCTCTTCGGAT |
|  | Reverse | AATCGGAGCCATAGTCCAATAG |
| H-CD9 | Forward | ACTGCGCGCTTCTAATTCCT |
|  | Reverse | CTGAGAGTCGAATCGGAGCC |
| m-AdipoQ | Forward | TGTTCCTCTTAATCCTGCCCA |
|  | Reverse | CCAACCTGCACAAGTTCCCTT |
| m-Fabp1 | Forward | GGAAGGACATCAAGGGGGTG |
|  | Reverse | TCACCTTCCAGCTTGACGAC |
| m-Fatp1 | Forward | ACTCTGCAAAGGGCTCATCC |
|  | Reverse | GCACGCATGCTGTAGGAATG |
| m-Apoa4 | Forward | GCCTGAGGGAGAAGGTCAAC |
|  | Reverse | CTCTCCAGAGGTTTGGGCTG |
| m-Mttp | Forward | CTCTTGGCAGTGCTTTTTCTCT |
|  | Reverse | GAGCTTGTATAGCCGCTCATT |
| m-Pparγ | Forward | TGGACACCATACTTGAGCAGA |
|  | Reverse | CAGGAGCAGAGCAAAGAGGT |
| H-Pparγ | Forward | ACAAGGCCATTTTCTCAAACGAG |
|  | Reverse | TGGGCGGTTGATTTGTCTGT |
| m-Srebp-1c | Forward | CACTTCTGGAGACATCGCAAAC |
|  | Reverse | ATGGTAGACAACAGCCGCATC |
| H-Srebp-1c | Forward | CAGGACAGGCAGAGGAAGAC |
|  | Reverse | TGAGGACAGCAAGGCAAAG |
| m-Acc1 | Forward | CGAAGGGCTTACATTGCCTA |
|  | Reverse | GGATGTTCCCTCTGTTTGGA |
| H-Acc1 | Forward | CCGCTTGCCTGACTTTTGAT |
|  | Reverse | GCCCTCCTTCTCCTCCAGTA |
| m-Scd1 | Forward | TGTCTCGGTGTGTGTCGGAGT |
|  | Reverse | TGTACCACTACCTGCCTGCATG |
| H-Scd1 | Forward | GCACCACAGCATATCGCAAG |
|  | Reverse | TCCAGAGGAGGTACTACAAACCT |
| m-Pparα | Forward | AGAGCCCCATCTGTCCTCTC |
|  | Reverse | ACTGGTAGTCTGCAAAACCAAA |
| H- Pparα | Forward | ACGATTCGACTCAAGCTGGT |
|  | Reverse | GTTGTGTGACATCCCGACAG |
| m-Fasn | Forward | AAGTTGCCCGAGTCAGAGAA |
|  | Reverse | GACCGCTTGGGTAATCCATA |
| H-Fasn | Forward | CAGAGCAGCCATGGAGGAG |
|  | Reverse | CTCAAGAACTGCACGGAGGT |
| m-Acadm | Forward | TGTGGAGAAGCTGATGAGGG |
|  | Reverse | TTTTCCCCTGAAGCAGCAAC |
| H-Acadm | Forward | TAATTGGTGACGGAGCTGGT |
|  | Reverse | GAATCAACCTCCCAAGCTGC |
| m-Acox1 | Forward | TAACTTCCTCACTCGAAGCCA |
|  | Reverse | AGTTCCATGACCCATCTCTGTC |
| H-Acox1 | Forward | AATCGGGACCCATAAGCCTTT |
|  | Reverse | GGGAATACGATGGTTGTCCATTT |
| m-Nox4 | Forward | TGCCTGCTCATTTGGCTGT |
|  | Reverse | CCGGCACATAGGTAAAAGGATG |
| H-Nox4 | Forward | TGTGCCGAACACTCTTGGC |
|  | Reverse | ACATGCACGCCTGAGAAAATA |
| m-CFD | Forward | TACATGGCTTCCGTGCAAGTG |
|  | Reverse | CACAGAGTCGTCATCCGTCA |
| H-CFD | Forward | GACACCATCGACCACGACC |
|  | Reverse | GCCACGTCGCAGAGAGTTC |
| m-Osgin1 | Forward | CCTCCGGTATCTGCCTGTC |
|  | Reverse | GGAAAGGTACTCTAGGTCCTGG |
| m-Car3 | Forward | CCAGCCACAATGGTCCTGAT |
|  | Reverse | AGTTTCTTGCCCGAAGAGGG |
| m-Tgfbr1 | Forward | TCTGCATTGCACTTATGCTGA |
|  | Reverse | AAAGGGCGATCTAGTGATGGA |
| m-Fgf21 | Forward | CAAGACACTGAAGCCCACCT |
|  | Reverse | TTGCATCCTGGTTTGGGGAG |
| m-Got1 | Forward | AAGCAGATCGCTGCTGTCAT |
|  | Reverse | AATAGCGAATAGCCCACGCA |
| m-Kidins220 | Forward | AGAGGGGCACATCCACATTG |
|  | Reverse | CTTGGGTTGGCACCATGAGA |
| m-Pbrm1 | Forward | TCATGCCCTATACACCCCCA |
|  | Reverse | AGGATATGGAGGTGGTGCCT |
| m-Lcp1 | Forward | ACTGAGAATTCAAGTCTGTCACCT |
|  | Reverse | CTATGCCATCGCCTACAGCA |
| m-Sirt1 | Forward | ACTGGAGCTGGGGTTTCT |
|  | Reverse | CTTGAGGGTCTGGGAGGT |
| m-Ucp2 | Forward | AAGTGTTTCGTCTCCCAGCC |
|  | Reverse | GGGACCTTCAATCGGCAAGA |
| H-Ucp2 | Forward | ACAAGACCATTGCCCGAGAG |
|  | Reverse | AGGAGGGCATGAACCCTTTG |
| m-Pgc-1α | Forward | TCTCAGTAAGGGGCTGGTTG |
|  | Reverse | TTCCGATTGGTCGCTACACC |
| H-Pgc-1α | Forward | TCCTTTGGGGTCTTTGAGAA |
|  | Reverse | GGCACGCAATCCTATTCATT |
| m-Cpt1a | Forward | TCAAGCCAGACGAAGAACATC |
|  | Reverse | TGGTAGGAGAGCAGCACCTT |
| H-Cpt1a | Forward | GCACATCGTCGTGTACCATC |
|  | Reverse | AATAGGCCTGACGACACCTG |
| m-Cidea | Forward | TGACATTCATGGGATTGCAGAC |
|  | Reverse | CATGGTTTGAAACTCGAAAAGGG |
| m-FLI1 | Forward | CAGTTACCTCAGGGAAAACCCTT |
|  | Reverse | TGGGGGAGGTCCAGTATTGT |
| H-FLI1 | Forward | GTGGATGGCAACTGGAAC |
|  | Reverse | ATGCCCTTGGCGTCTTA |
| m-18s rRNA | Forward | AGGGGTTCGGGATTTGTG |
|  | Reverse | GACCAGGCGGAACAGAGA |
| m-Gapdh | Forward | TGAACGGGAAGCTCACTG |
|  | Reverse | TCCACCACCCTGTTGCTG |
| H-Gapdh | Forward | GGGTGTGAACCACGAGAAAT |
|  | Reverse | CCTTCCACAATGCCAAAGTT |
| ChIP-CFD  -990 to -795bp | Forward | TTGTAGAGATGGGGTCTCGCCG |
|  | Reverse | AGCCAAGTCTCAAGGGTGCC |
| ChIP-CFD  -789 to -600bp | Forward | TGTGATCCAGGCTCCACCAAC |
|  | Reverse | TTCAGGGATGTCCGCCTTCAG |
| ChIP-CFD  -606 to -585bp | Forward | ACCTGGATCAAGCCACACCTG |
|  | Reverse | AGGTCTGTTGGGGGTAGGAAG |
| ChIP-CFD  -414 to -221bp | Forward | AAGTGACCTTCCTACCCCCAAC |
|  | Reverse | GCAGTCCCTCAGATGGGCA |
| ChIP-CFD  -203 to +18bp | Forward | TGCCTCCTGCCCATCTGAGG |
|  | Reverse | GGTGAAGCCGCTGTGGC |
